# Biomass-Derived Solvents and Low-GWP Refrigerants as Working Fluids for Sustainable Absorption Refrigeration

**DOI:** 10.1021/acssuschemeng.5c00258

**Published:** 2025-05-19

**Authors:** Miguel Viar, Fernando Pardo, Gabriel Zarca, Ane Urtiaga

**Affiliations:** Department of Chemical and Biomolecular Engineering, 16761 Universidad de Cantabria, Av. Los Castros 46, Santander 39005, Spain

**Keywords:** hydrofluoroolefins, green solvents, biobased, solubility, refrigeration cycle

## Abstract

A shift toward more sustainable practices is critical for the refrigeration, air conditioning, and heat pump (RACHP) sector, which is responsible for significant greenhouse gas emissions due to its reliance on vapor compression refrigeration cycles. Absorption refrigeration systems (ARS) have been proposed as a promising alternative due to their ecofriendliness, especially when powered by low-grade heat. This work introduces a novel approach by incorporating eco-friendly and biobased solvents as working fluids in ARS for the first time. Five green organic solvents—solketal, propylene carbonate, terpinolene, γ-valerolactone, and Rhodiasolv PolarClean—were carefully selected based on their safety, operational, and environmental profiles, assessed by referring to the CHEM21 solvent selection guide. Subsequently, the affinity and interactions between these solvents and three hydrofluorocarbons (HFCs): R-32, R-125 and R-134a, and two hydrofluoroolefins (HFOs): R-1234yf and R-1234ze­(E), were assessed using COSMO-RS quantum chemical calculations. The vapor–liquid equilibrium (VLE) of the binary systems was experimentally determined at several temperatures and pressures, followed by an in-depth thermodynamic evaluation to select the most promising solvent-refrigerant pairs. Finally, the coefficient of performance (COP) and the circulation factor (*f*) of γ-valerolactone and Rhodiasolv PolarClean-based working pairs were evaluated within the ARS framework, showcasing a significant breakthrough in the development of R-1234ze­(E)-based pairs. Notably, the pairs including R-1234ze­(E) achieved the highest COP value (0.60) reported to date with HFOs in analogous ARS. Moreover, the compression-assisted ARS (CA-ARS) evaluated proved to be competitive in terms of COP and *f* when compared to those of conventional pairs. These results highlight the promising potential of green organic solvents and low-GWP HFC/HFO-based working pairs as an effective strategy for reducing emissions and improving the sustainability of the RACHP sector.

## Graphical abstract

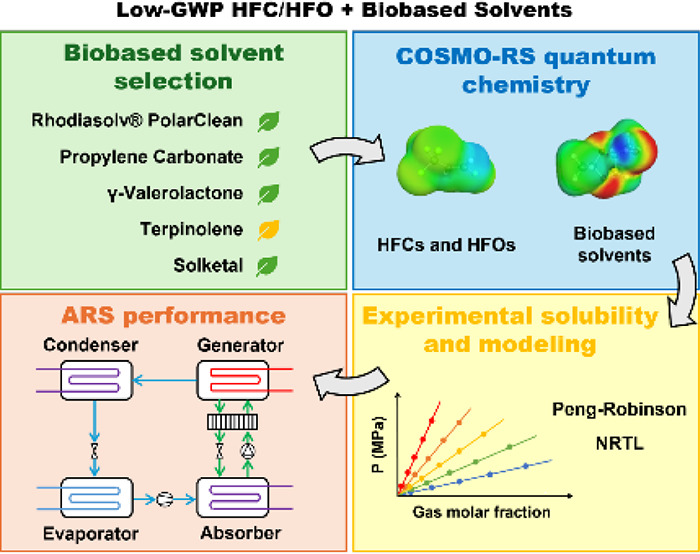

## Introduction

Climate change is one of the most pressing contemporary concerns, driven largely by energy production from nonrenewable sources; this accounted for about 65% of all greenhouse gas (GHG) emissions in 2023.^1^ To address this issue, efforts are being focused on both promoting the use of renewable energy sources and decarbonizing industrial processes. This is particularly relevant in the refrigeration, air conditioning, and heat pump (RACHP) sector, where energy-intensive technologies are applied to fulfill their vital role in sustaining human life, including applications such as the preservation of food and medicines, as well as ensuring human comfort in zones with warm and hot temperatures.^2,3^ The exponential growth of the RACHP sector could account for up to 20% of direct and indirect emissions of GHGs by the mid-21st century, highlighting the urgent need to pursue more energy-efficient and greener refrigeration technologies.^3–5^ In this context, absorption refrigeration systems (ARS) are emerging as a promising alternative to conventional vapor compression refrigeration (VCR) in large stationary and industrial applications.^6^ In particular, ARS offer two key advantages over VCR: (i) a reduced energy footprint, as the compressor used in VCR is replaced by an absorption–desorption cycle; and (ii) the possibility of using a low-grade heat source for desorption in the generator.^7^ Considering that approximately 72% of global energy consumption is lost as waste heat, ARS, therefore, presents an energy efficiency and sustainability-enhancing opportunity in the RACHP sector.^1,6^

Working pair selection is a key factor that directly impacts process performance and, therefore, the long-term sustainability of ARS.^4,7^ The conventional refrigerant/absorbent pairs used in ARS are H_2_O/LiBr and NH_3_/H_2_O. Both deliver high performance but also pose operational, environmental, and economic challenges. The H_2_O/LiBr system faces crystallization and corrosion issues that reduce equipment lifespan, whereas the NH_3_/H_2_O system requires a rectifier after the desorption stage due to the complexity of its separation and is not risk-free in terms of safety should there be an accidental release of the working fluid.^4,8^ These limitations highlight the urgent need to develop alternative working pairs designed to meet sustainability goals.

In this context, ionic liquid (IL)-based working pairs have emerged as promising alternatives to traditional absorption pairs due to the exceptional properties of IL absorbents, such as negligible vapor pressure, high chemical and thermal stability, and excellent solubility for both polar and nonpolar refrigerants.^4,9^ Research to date has primarily focused on binary IL-based working pairs, including H_2_O/IL, NH_3_/IL, CO_2_/IL, alcohol/IL, and HFC or HFO/IL.^4^

The choice of refrigerant is critical, as this directly influences the operating conditions and performance of absorption refrigeration systems (ARS).^10^ While H_2_O- and NH_3_-based pairs exhibit similar limitations to conventional mixtures, CO_2_ and alcohol refrigerants require significantly higher operating pressures and pose flammability risks, necessitating stricter safety measures.^10^ Consequently, hydrofluorocarbons (HFCs) and hydrofluoroolefins (HFOs) have gained attention as viable alternatives when paired with ILs, with extensive studies focusing on their solubility and performance in ARS.^3,10,11^ In this regard, Wu et al. assessed the behavior of HFCs (R-134a, R-32, R-152a, and R-161) and HFOs (R-1234yf and R-1234ze­(E)) in combination with the IL [C_6_C_1_im]­[Tf_2_N] in ARS.^12,13^ Using the same IL and refrigerants R-32, R-152a, R-1234yf, and R-1234ze­(E), Sujatha and Venkatarathnam analyzed the impact of ARS operating temperatures, achieving COP values of up to 0.5 with R-32.^14^ Moreover, due to the high viscosity of [C_6_C_1_im]­[Tf_2_N], Asensio-Delgado et al.^11^ proposed and analyzed the ARS performance of R-32, R-134a, R-1234yf, and R-1234ze­(E) paired with low-viscosity ILs, such as [C_2_C_1_im]­[BF_4_], [C_2_C_1_im]­[OTf], [C_2_C_1_im]­[SCN], and [C_2_C_1_im]­[Tf_2_N], achieving COP values close to 0.8 in the best-case scenario. Additionally, Moreno et al.^15^ and Zhang et al.^10^ applied COSMO-RS to predict the thermodynamic properties of working pairs and used Aspen simulations to evaluate the ARS performance of the most promising low-GWP refrigerant/IL combinations.

There are, however, major concerns about the use of ILs, particularly in terms of their high viscosity, elevated market prices, and potential environmental issues related to their synthesis, biodegradability, and toxicity.^16^ It is, therefore, necessary to find alternative solvents that can compete with ILs but have an improved sustainability profile. The use of solvents that adhere to green chemistry principles would also result in safer, eco-friendly, and more sustainable absorption refrigeration.^17^ These principles underscore the utilization of solvents derived from renewable feedstocks, which are widely available, minimally toxic, highly biodegradable, nonflammable, thermally and chemically stable, and cost-effective.^17–19^ Thus, it can be reasonably deduced that biomass-derived solvents may represent a significant advance in the development of more eco-friendly and efficient ARS. For this purpose, solvent selection guides, such as CHEM21, can be an effective and accessible tool for evaluating the Environmental, Health, and Safety (EHS) profile of the solvents.^20–22^ This kind of guide qualifies certain organic solvents proposed for use in ARS,^8^ such as *N*-methyl-2-pyrrolidone (NMP), dimethylformamide (DMF), and dimethylacetamide (DMAC), as problematic or not recommended for use due to their toxicity and environmental issues.^20,22^

This paper, therefore, presents a novel approach by proposing, for the first time, the use of green organic solvents as an alternative to ILs, coupled with low-GWP HFCs and HFOs, to enhance the sustainability footprint of ARS. A rational method was followed to identify the most suitable working pairs. First, five organic solvents were selected based on their favorable physicochemical properties and potential biobased synthesis routes, with their green nature being preliminarily assessed using the CHEM21 selection guide. Next, we analyzed the affinity and interactions of the solvents with three HFCs (difluoromethane, pentafluoroethane, and 1,1,1,2-tetrafluoroethane) and two HFOs (2,3,3,3-tetrafluoropropene, trans-1,3,3,3-tetrafluoropropene) using COSMO-RS quantum chemistry calculations, and we experimentally determined the solubility of each pair across a range of temperatures and pressures. The vapor–liquid equilibrium (VLE) was modeled using the non-random two-liquid (NRTL) activity coefficient model and the Peng–Robinson equation of state. Finally, we selected the most promising solvent-HFC/HFO pairs and assessed their performance in two ARS configurations: single-effect and compression-assisted ARS. This research contributes to broadening the scope of sustainable solutions by shifting the focus from IL-based working pairs to renewable-based solvents. It integrates computational modeling with experimental validation to provide a robust framework for selecting new ARS working pairs.

## Experimental Section

### Materials

The refrigerant gases difluoromethane (R-32), pentafluoroethane (R-125), 1,1,1,2-tetrafluoroethane (R-134a), 2,3,3,3-tetrafluoropropene (R-1234yf), and trans-1,3,3,3-tetrafluoropropene (R-1234ze (E)) were supplied by Coproven Climatización (a Gas Servei licensed supplier, Spain) with a purity higher than 99.9 vol %. Table 1 provides an overview of the main properties of these refrigerants.

**Table 1:** F-Gases Used in This Work

Refrigerant	Formula	CAS No.	Molecular weight (g·mol^–1^)	Boiling point (K)	GWP_100_ (kg CO_2_-eq/kg)
Hydrofluorocarbons (HFCs)					
R-32	CH_2_F_2_	75-10-2	52.02	221.45	771
R-125	C_2_HF_5_	354-33-6	120.02	224.65	3740
R-134a	C_2_H_2_F_4_	811-97-2	102.03	247.08	1260
Hydrofluoroolefins (HFOs)					
R-1234yf	C_3_H_2_F_4_	754-12-1	114.04	243.67	0.501
R-1234ze(E)	C_3_H_2_F_4_	29118-24-9	114.04	254.19	1.37

The solvents used in this work were solketal (Apollo Scientific, >99.0 vol %), propylene carbonate (Thermo Scientific, >99.0 vol %), terpinolene (Merck, >95.0 vol %), γ-valerolactone (Merck, >99.0 vol %), and Rhodiasolv PolarClean (Solvay, >95.0 vol %). Table 2 lists some of the solvent properties relevant to the present study.

**Table 2:**
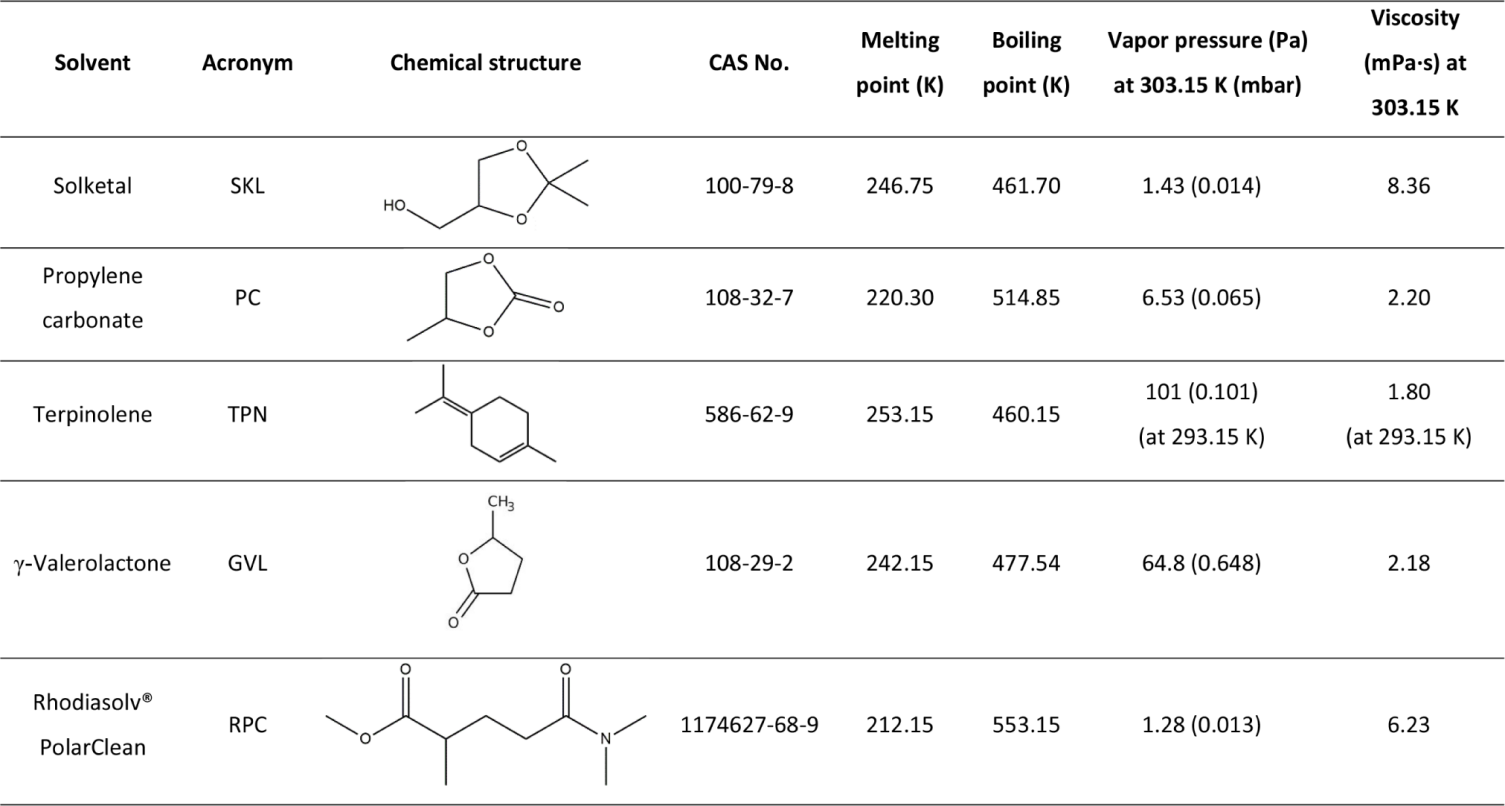
Properties of the Selected Organic Solvents.^24–31^

The heat capacity of Rhodiasolv PolarClean was determined within the temperature range of 298.15–358.15 K using a Calvet calorimeter (Setaram model C-80, ± 1 J K^–1^ mol^–1^). The experimental procedure is detailed in the Supporting Information, where Table S1 compiles the heat capacity data for Rhodiasolv PolarClean obtained in this study with the heat capacity of γ-valerolactone as reported by Nikitin et al.^23^ Both data sets were utilized in the ARS calculations.

### COSMO-RS Calculations

The COSMO-RS method was implemented by using COSMOtherm software V2024. All of the compounds studied were available in the COSMObase V24 database with TZVPD-FINE parametrization. The software provides the probability of a particular charge density on a surface segment as a σ-profile, allowing us to interpret the interactions and affinities between molecules.^32^ It also enabled us to predict the VLE and Henry’s law constant between the fluorinated gases and the solvents.^33–35^

### Experimental Solubility Measurement and Modeling

The isochoric saturation method was applied to determine the solubility of each refrigerant gas in the selected solvents. The apparatus used and the experimental procedure followed have been described in detail in our previous publications.^36–38^ The experimental setup consisted of a jacketed stirred tank reactor (Buchi, Picoclave model, 170 mL), equipped with a pressure transducer (Keller, PAA-33X series, 0.01% accuracy) and a Pt-100 temperature sensor connected to a thermostatic bath (Grant, model LT ecocool 150, ± 0.01 K). The reactor was connected to the storage cylinder (140 mL) by a valve and was equipped with another pressure transducer.

Approximately 30 g (±0.0001 g) of solvent was loaded into the absorption chamber, ensuring that the gas volume introduced was greater than that of the solvent to minimize the effect of its volumetric expansion during gas absorption. Prior to each experiment, the solvent was regenerated under high-temperature and vacuum conditions. After the working temperature of the experiment was adjusted, a certain amount of gas was introduced into the storage cylinder, and the pressure and temperature were recorded. The connection valve was then opened, allowing the gas and solvent phases to come into direct contact within the absorption chamber. The stirrer was set to a speed of 500 rpm to facilitate the absorption process, and the pressure and temperature were continuously recorded until equilibrium was achieved; i.e., a constant pressure was maintained for more than 20 min.

The experimental procedure and its validation with the CO_2_-propylene carbonate system are included in this work as Supporting Information. Two approaches were used to model the experimental solubility data: (i) the non-random two-liquid (NRTL) model, based on activity coefficients; and (ii) the Peng–Robinson equation of state coupled with the Boston–Mathias mixing rule (PR-BM). A detailed description of these two approaches can also be found in the Supporting Information.

### Absorption Refrigeration Modeling

Figure 1 depicts the two ARS schemes that were evaluated. In the single-effect ARS (SE-ARS), the liquid phase formed by the absorbed refrigerant and the solvent is pumped to the generator, where the refrigerant is desorbed, preferably using heat sourced from renewable energy or waste heat. Subsequently, the refrigerant is condensed at high pressure, expanded, and directed to the evaporator, where it absorbs heat from its surroundings to generate the desired cooling effect. Finally, the refrigerant is reabsorbed by the solvent, completing the cycle.^8^ In the compression-assisted ARS (CA-ARS), a small compressor is installed prior to the absorber to increase the refrigerant pressure and thus enhance its solubility in the solvent.^11^ The mathematical modeling of the ARS is outlined in detail in the Supporting Information. The model was run in MATLAB software, following a previously validated procedure.^11^

**Figure 1:**
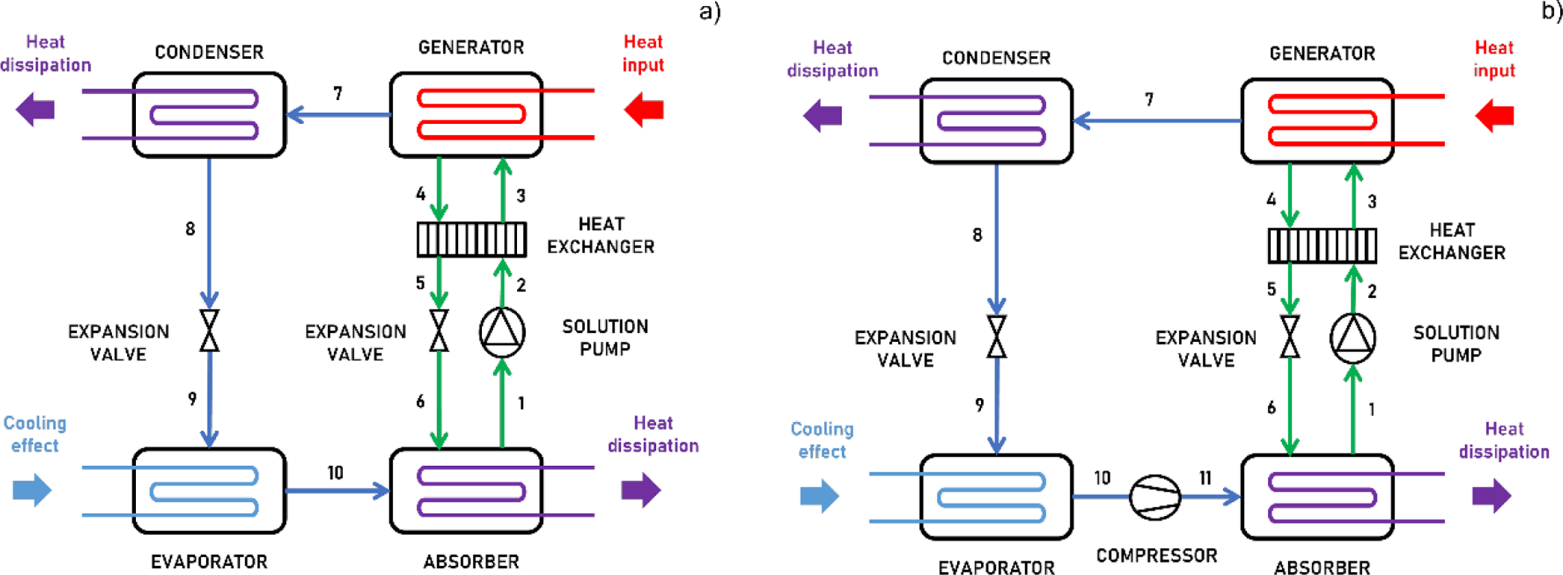
Schematic diagram of: (a) SE-ARS and (b) CA-ARS. Each stream is numbered to facilitate understanding of the energy balances outlined in the Supporting Information.

## Rational Selection of Greener Absorbents for ARS

We based the selection of green organic solvents as potential absorbents of fluorinated refrigerants for ARS on a multifaceted approach that takes into account the following criteria in terms of solvent properties: (i) a low melting point to ensure operation in the liquid state; (ii) a high boiling point and low vapor pressure to ease regeneration and avoid solvent impurities in the vapor streams; (iii) low viscosity for improved mass and heat transfer rates; and (iv) an assessment of their green nature. It is worth noting that solketal, terpinolene, γ-valerolactone, and Rhodiasolv PolarClean can be synthesized via biobased routes,^17,39–42^ emphasizing their biomass origin. In particular, solketal is derived from the catalytic processing of glycerol, a byproduct of the transesterification of natural fats (triglycerides);^39^ terpinolene, a naturally occurring monoterpene, is found in the essential oils of various aromatic plant species;^43^ γ-valerolactone is synthesized via the hydrogenation of levulinic acid, which is produced through the hydrolysis of cellulose, starch, and hemicellulose;^44^ and Rhodiasolv PolarClean originates from agrochemical formulations.^45^ In contrast, propylene carbonate cannot be derived from biomass, although ongoing research is focused on developing a synthesis process in line with green chemistry principles, including the possibility that captured CO_2_ may be used as a reactant.^46,47^

Given the low melting points, vapor pressures, and high boiling points of the five solvents (Table 2), these would remain in the liquid phase throughout the absorption–desorption cycle, fulfilling selection criteria (i) and (ii) above. Employing HFCs and HFOs as refrigerants, whose boiling points are lower than 254.15 K, widens the difference in boiling points between the refrigerant and the solvent, another requirement for ARS. The viscosity of all the solvents is notably low (criteria (iii)), especially when compared to the ILs investigated in other studies—a feature that is likely to improve mass and heat transfer rates and, therefore, the overall efficiency of ARS in terms of both operational and economic performance. Compared to commercial solvents, the viscosity of solketal is only slightly higher than that of the LiBr/H_2_O system (7 cP for a 0.60 kg/kg LiBr/H_2_O solution^48^) , and both types are more viscous than the H_2_O/NH_3_ system (0.80 cP^49^).

Next, the environmental profile of each solvent was analyzed (criteria (iv)) using the CHEM21 solvent selection guide,^20,21^ which evaluates the physicochemical properties of compounds, such as their boiling, flash, and autoignition points, as well as their potential health and environmental risks, in line with the Globally Harmonized System (GHS) for classifying and labeling chemicals.^21,50^ According to these properties, each solvent receives a score from 1 to 9 in categories related to environmental, health, and safety criteria, with lower scores indicating greener, more environmentally favorable solvents. Based on these scores, the solvents are further classified as recommended (green), problematic (yellow), or hazardous (red).^21,22^ In this work, an adjustment was made regarding the effect of the solvent boiling point on the environmental score. The reason for this is that the CHEM21 framework, originally developed for the pharmaceutical industry, penalizes solvents with a high boiling point, as they would result in greater energy consumption during the recovery stage, thereby conferring a less favorable environmental score. In ARS, however, refrigerant desorption is achieved at relatively high temperatures and low pressures.^51^ Accordingly, a solvent with a high boiling point is advantageous for ARS, as this would prevent its transition to the gas phase and the presence of impurities. Finally, the boiling point was evaluated in accordance with three criteria. Solvents with boiling points below 100 °C were assigned the worst score of 7, as they were deemed unsuitable for ARS;^52^ solvents with boiling points above 150 °C received the greenest score of 3, as they would remain in the liquid phase regardless of the generation temperature or ARS design, which have mainly been investigated from 20 to 150 °C to date.^8^ Solvents with boiling points between these values were given an intermediate score of 5.

The results of the EHS assessment in Table 3 indicate that the solvents considered in this study are rated as recommended, except for terpinolene, which was categorized as problematic. This latter result is mainly due to the adverse impact that a potential release of terpinolene into the marine environment would have on aquatic life. Following the CHEM21 guidelines, any industrial process using terpinolene should implement measures to prevent its discharge into water bodies.^22^ Thus, considering that the scope of this work is confined to ARS circuits and given the biobased origin of terpinolene, this solvent was included in the analysis.

**Table 3:**
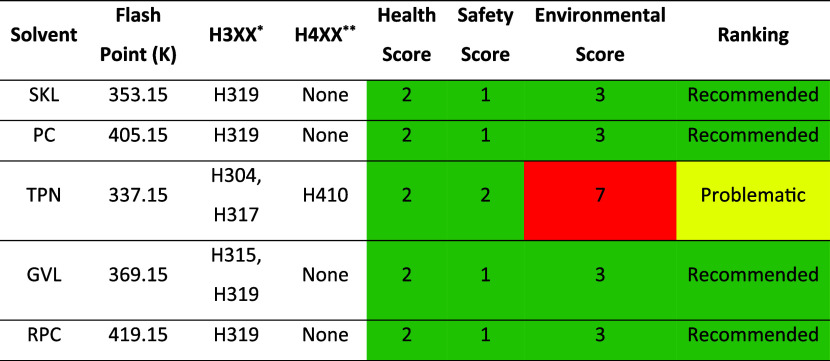
EHS Assessment of Each Solvent Under Study Following the CHEM21 Guide Adapted to Select Solvents for ARS^a^

a H3XX*: hazard statements for health hazards. H304: may be fatal if swallowed and enters airways; H315: causes skin irritation; H317: may cause an allergic skin reaction; and H319: causes serious eye irritation. H4XX**: hazard statements for environmental hazards. H410: very toxic to aquatic life with long lasting effects.

## Results and Discussion

### Refrigerant–Absorbent Affinity

The initial step in determining the suitability of a working pair for use in ARS is to assess the affinity between the refrigerant and the absorbent. For this, the solvation environment of the fluorinated refrigerants in the selected solvents was analyzed. The COSMO-RS software enables a predictive approach by defining the chemical structure and conformers of the substances. The σ-profile allows an assessment of the affinity between different refrigerant gas/solvent pairs by showing the polarity of the molecules according to the charge distribution on their molecular surface. The σ-profiles of refrigerants and solvents (shown in Figure 2) can be divided into three sections: the hydrogen bond donor (HBD) region (σ < −0.0082 e/Å²); the nonpolar region (−0.0082 e/Å^2^<σ < 0.0082 e/Å^2^); and the hydrogen bond acceptor (HBA) region (0.0082 e/Å^2^< σ).^10,53^ Figure 2a shows the σ-profiles of the HFC and HFO refrigerants considered in this work. They were mainly nonpolar molecules with a charge distribution around σ = 0^+^, but they also exhibited small peaks in the HBD region. This was mainly attributed to the high electronegativity of the fluorine atoms, which induces a charge defect on the hydrogen atoms, giving them a more polar character.^15^ Thus, the HBD character of the fluorinated refrigerants followed the order R-125 > R-1234ze­(E) > R-134a > R-32 > R-1234yf. In view of their mainly nonpolar and slightly HBD character, the solvents with the greatest affinity for the selected refrigerants would be those that exhibit complementary peaks in the nonpolar region, with a slightly polar character in the HBA region. Figure 2b shows the charge distributions of the solvents studied. They all had a predominantly nonpolar character. In particular, terpinolene was completely nonpolar according to its hydrocarbon molecular structure, with no functional groups. Its entire charge distribution is in the nonpolar region, coinciding with that of the fluorinated gases, indicating terpinolene’s reduced affinity for the gases studied. However, the other four solvents exhibited a large nonpolar area, distributed near to σ = 0^–^, with complementary peaks to the nonpolar peaks of the refrigerant gases, distributed around σ = 0^+^. Additionally, the presence of oxygenated groups in the molecular structure of the four solvents, and an amino group in Rhodiasolv PolarClean, gave them a slight HBA character,^32^ leading to a great affinity between the fluorinated refrigerant gases R-125, R-1234ze­(E), R-134a, R-32 and R-1234yf and the solvents solketal, propylene carbonate, γ-valerolactone, and Rhodiasolv PolarClean. Overall, these four solvents were likely to present excellent HFC and HFO solubility.

**Figure 2:**
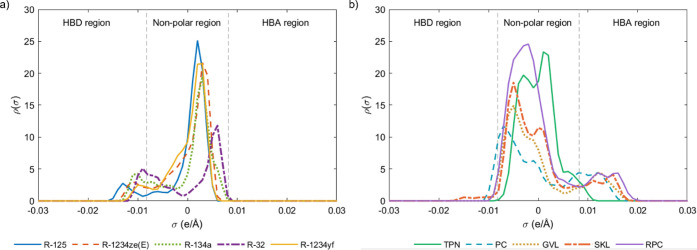
σ-profile of the: (a) fluorinated refrigerant gases and (b) solvents used in this work.

### HFC and HFO Solubility in Green Solvents

To validate the predictions achieved through the quantum chemistry calculations, the solubility of R-32, R-125 and R-134a, R-1234yf, and R-1234ze­(E) was experimentally determined in solketal, propylene carbonate, terpinolene, γ-valerolactone, and Rhodiasolv PolarClean over a temperature range of 283.15–323.15 K and pressures up to 1 MPa. The empirical VLE data for all the refrigerant gas/green solvent pairs are compiled in Tables S3–S15. Tables S16 and S17 present the optimized fitting parameters of the NRTL and PR-BM thermodynamic models. As representative examples, Figures 3 and 4 show the absorption isotherms of the three HFCs, R-32, R-125 and R-134a, and the two HFOs, R-1234yf and R-1234ze­(E), in γ-valerolactone and Rhodiasolv PolarClean. As can be seen, the NRTL and, especially, the PR-BM models described the solubility of HFCs and HFOs in the solvents particularly well. The high degree of accuracy of the two models was also observed for the other solvents, as demonstrated by the low average absolute relative deviation (AARD) values between the experimental and simulated solubility data reported in Tables S16 and S17. Comparing the two approaches, only two parameters had to be adjusted in the NRTL model in most cases, whereas this number was at least four for the PR-BM model, hence the decreased deviations observed, particularly at the highest pressures tested. This was expected given that PR-BM is an equation of state (EoS), whereas the NRTL model is an activity coefficient model whose applicability is usually limited to the low-pressure range. In addition, the VLE data were also predicted by the COSMO-RS method. Figures S2 and S6 compare the experimental and predicted COSMO-RS data for each solvent/gas pair. As can be seen, COSMO-RS was able to qualitatively predict the absorption trend, although it was still far from achieving a quantitative prediction, exhibiting AARDs between the predictions and experimental data ranging between 8.9% for the R-32/Rhodiasolv PolarClean pair and 166.0% for the R-125/terpinolene pair.

**Figure 3:**
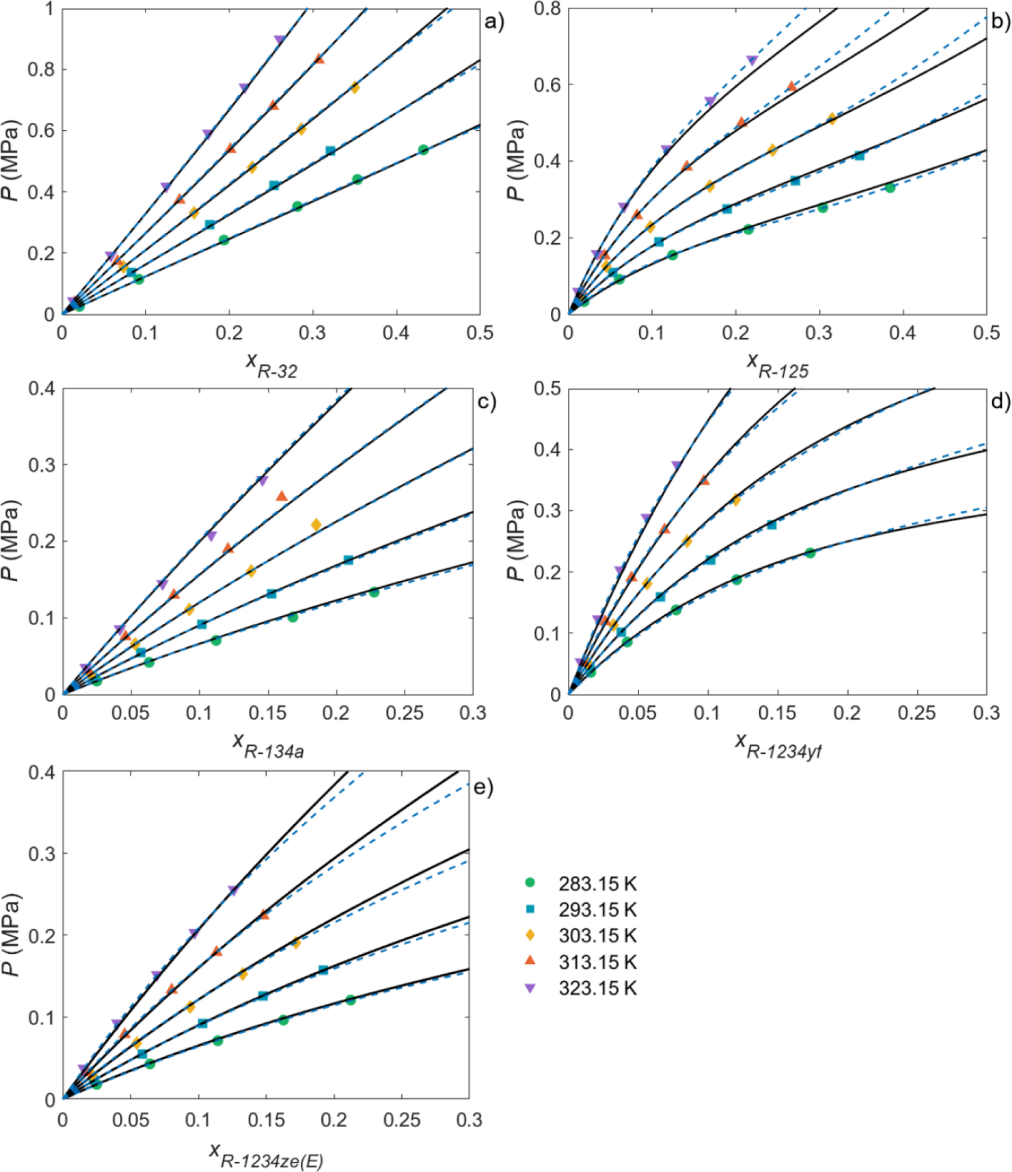
Experimental solubility data for (a) R-32, (b) R-125, (c) R-134a, (d) R-1234yf, and (e) R-1234ze­(E) in γ-valerolactone at different temperatures. The dashed and dotted lines represent the NRTL and PR-BM model calculations, respectively.

**Figure 4:**
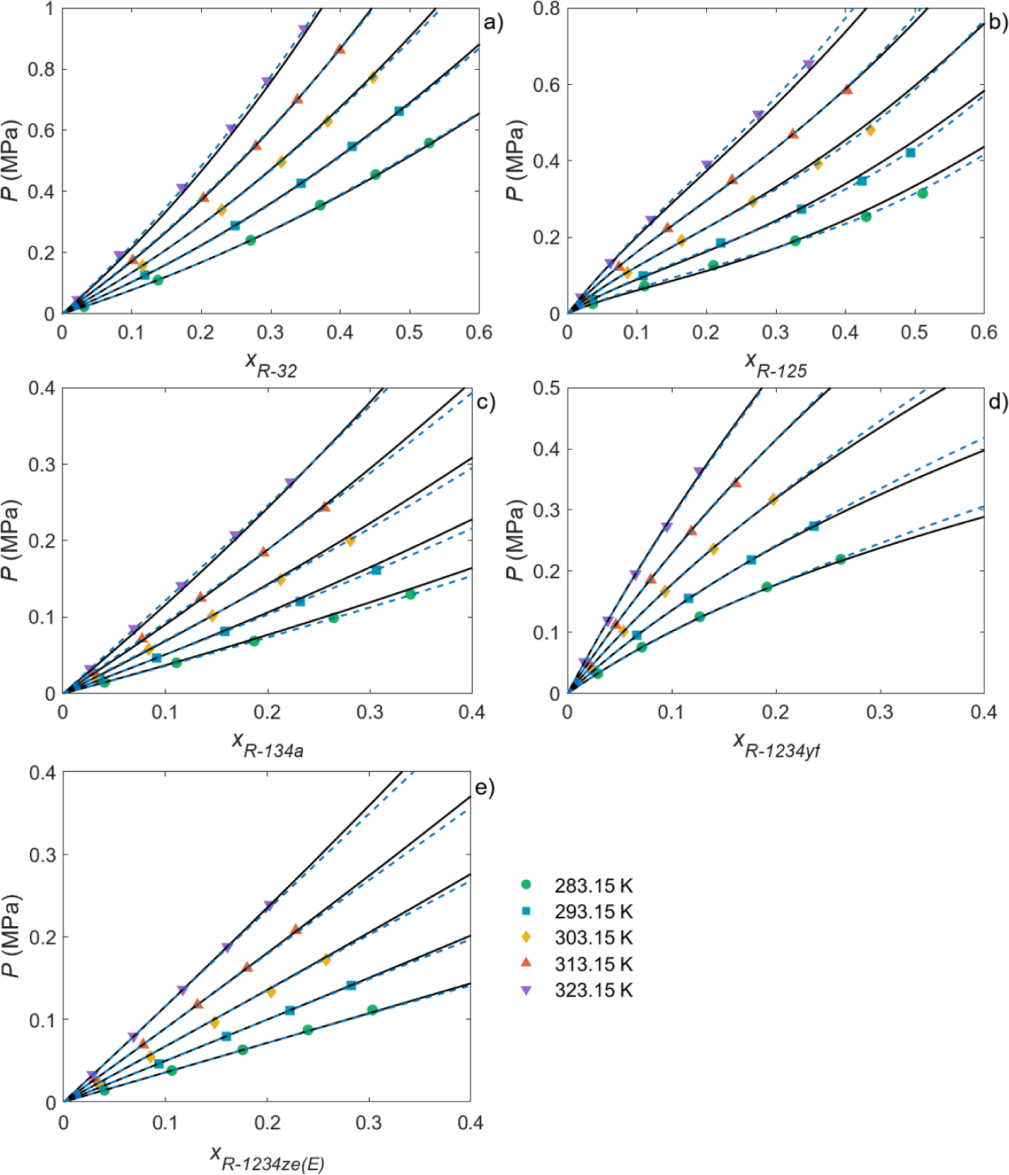
Experimental solubility data for (a) R-32, (b) R-125, (c) R-134a, (d) R-1234yf, and (e) R-1234ze­(E) in Rhodiasolv PolarClean at different temperatures. The dashed and dotted lines represent the NRTL and PR-BM model calculations, respectively.

In an ARS, it is fundamental to employ a solvent with high solubility toward the compound acting as a refrigerant. A comparison between all the working pairs assessed in this work can be easily performed by evaluating the Henry’s law constant (*k*_H_), eq 1.

1 $$k_{H}\left(T\right)=\underset{x\to 0}{\lim}\frac{\mathrm{f̅}\left(P,T\right)}{x}$$

where *f̅* is the refrigerant gas fugacity, and *x* is the refrigerant molar fraction in the liquid phase. To ascertain the limit at infinite dilution with eq 1, the experimental solubility was first fit to a second-order polynomial.^36^ Figure 5 depicts the experimental Henry’s law constants at 303.15 K for each solvent/refrigerant pair. In addition, Tables S18–S22 collect the values obtained from both the experimental data and the COSMO-RS method. In terms of the experimental outcomes for all the systems at low pressure, the COSMO-RS results were reasonably similar to the predicted Henry’s law constants, substantiating its utility as a valuable tool for screening suitable solvents for ARS in a preliminary assessment.

**Figure 5:**
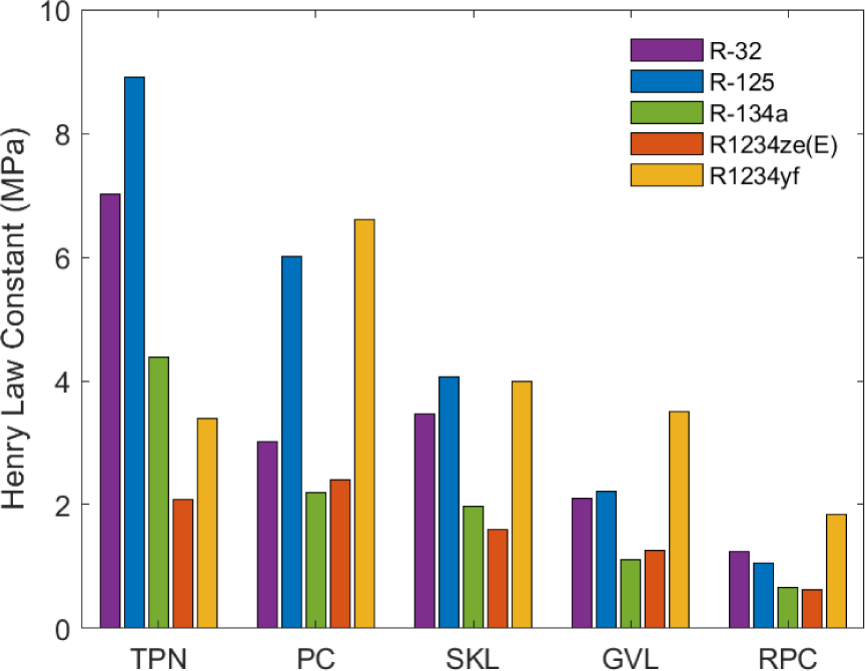
Henry’s law constants of F-gases and green solvents at 303.15 K.

Henry’s law constants provide insight into the solubility of a gas in a liquid. A lower value indicates a greater solubility of the gas in the solvent. Rhodiasolv PolarClean was the solvent with the highest absorption capacity for HFCs and HFOs, followed by γ-valerolactone and solketal. Terpinolene exhibited the lowest capacity for HFC dissolution, while HFOs exhibited a comparatively reduced solubility in propylene carbonate. R-1234ze­(E) and R-134a were the most soluble refrigerant gases. These results can also be compared with the experimental solubility data. For example, at a given temperature and pressure, the solubility of the gases in γ-valerolactone (Figure 3) followed the order: R-134a > R-1234ze­(E) > R-32 > R-125 > R-1234yf, i.e., the same trend as shown in Figure 5.

To gain further insight into refrigerant gas absorption in the green organic solvents studied, the enthalpy and entropy values of the solvation process were calculated following the procedure described in Section S10. The results are presented in Table S23 and Figure S7, showing that the absorption process was exothermic, in agreement with those reported in the literature.^3^ The range of enthalpy values, between 16.4 and 26.1 kJ/mol, indicates that in all cases, gas absorption by the solvents followed a physical mechanism, which would reduce the energy requirements of the desorption stage of the ARS. Regarding the solvents, the terpinolene pairs exhibited very close solvation enthalpies for the different refrigerants, being approximately 20% lower than the enthalpies observed with the other solvents, reflecting the reduced solubility and lack of strong molecular interactions between HFCs/HFOs and terpinolene. This finding agrees with the results of the COSMO-RS σ-profiles.

The potential use of green organic solvents and low-GWP HFC/HFOs as working fluids was first assessed based on their physicochemical properties and solubilities. In terms of their high solubility toward HFCs and HFOs, low viscosity, high boiling point, and low solvation enthalpy, which facilitates their desorption in the generator stage, the green organic solvents studied were qualified as potentially suitable for their use in ARS. The performance of the most promising working pairs is reported in the following section.

### ARS Performance

As a preliminary approach for evaluating the ARS performance with green solvents, two classical ARS configurations were selected: SE-ARS and CA-ARS. The ARS case study was defined by setting the temperatures of both the condenser (*T*_c_) and the absorber (*T*_a_) to 303.15 K, and the evaporator (*T*_e_) to 278.15 K, as a representative example of a refrigeration system.^11^ For CA-ARS, the compression ratio (CR) was set at 1.5. The main pairs selected were those formed from HFC R-134a and HFO R-1234ze­(E) with γ-valerolactone and Rhodiasolv PolarClean due to their high solubility. R-32 was also considered, as it is the HFC with the lowest GWP and had previously demonstrated favorable outcomes in ARS studies with alternative IL solvents.^13,14^

To evaluate the feasibility of the proposed absorption pairs based on green organic solvents, two performance indicators were calculated: (i) the coefficient of performance (COP) and (ii) the circulation factor (*f*), both defined in Section S4. The COP has traditionally been regarded as the most important parameter for evaluating ARS performance, defined as the ratio of the cooling power delivered by the process to the energy input required for its operation. The typical cooling COP of ARS is between 0.5 and 0.9.^13^ In contrast, *f* provides valuable insight into the amount of solvent needed in the ARS circuit. Figure 6 illustrates the COP and *f* values obtained for each absorption pair as a function of the generation temperature (*T*_g_), which corresponds to the temperatures of streams 4 and 7 in Figure 1 and the specific ARS configuration under study.

**Figure 6:**
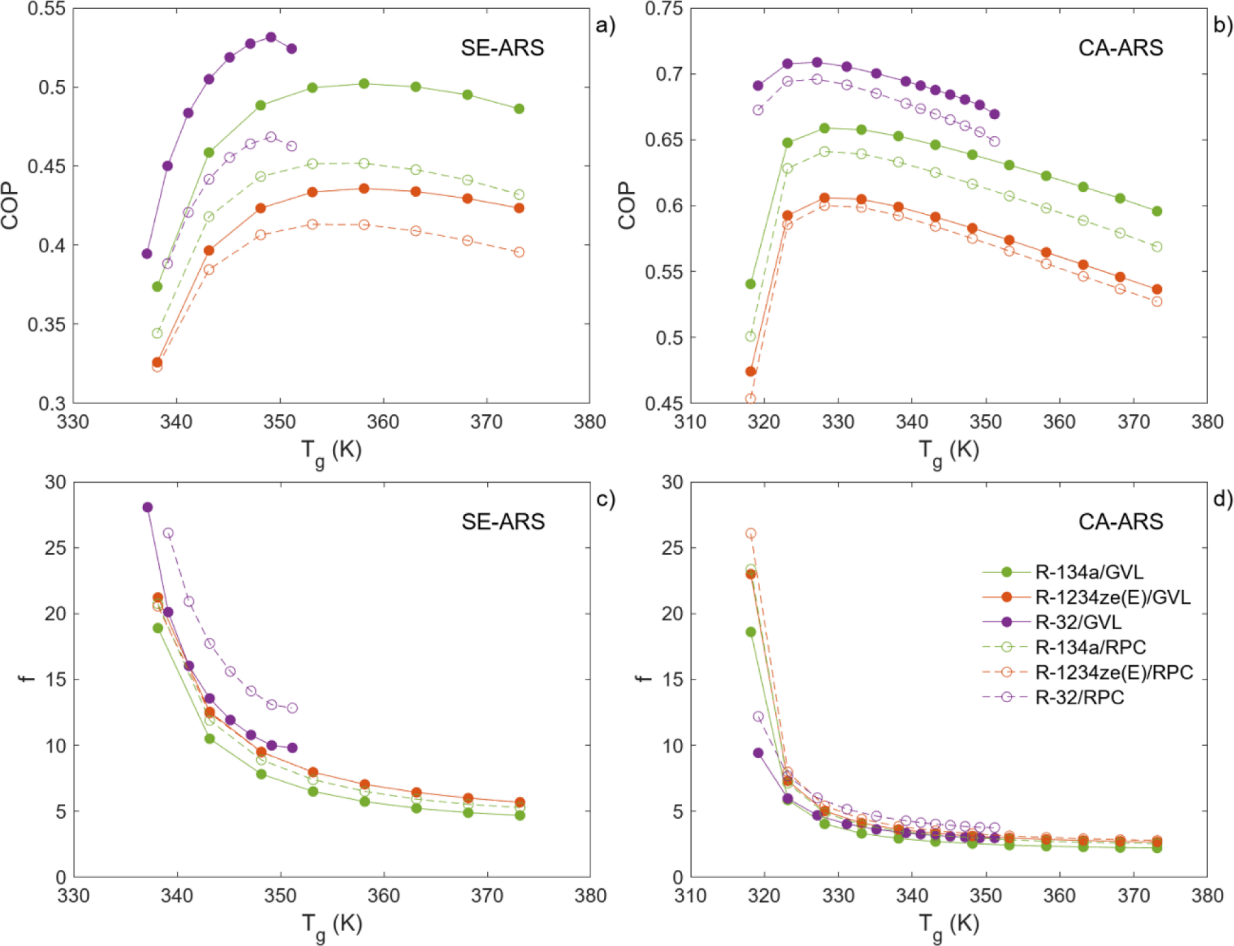
COP and *f* for SE-ARS (left) and CA-ARS (right) as a function of the generation temperature (*T*_a_/*T*_c_/*T*_e_ = 303.15/303.15/278.15 K, CR = 1.5). The solid lines correspond to the pairs formed using γ-valerolactone as a solvent, while the dashed lines correspond to the use of Rhodiasolv PolarClean as a solvent.

With regard to the SE-ARS (Figure 6a,c), γ-valerolactone exhibited slightly superior outcomes in terms of COP and *f* in comparison to Rhodiasolv PolarClean. In both cases, the trend of the curves exhibited a similar pattern, with the maximum COP values occurring at a *T*_g_ between 350 and 360 K. Despite its lower solubility compared to the other two gases, R-32 produced the most favorable results, reaching a maximum COP of 0.53 with an *f* of 10. The next refrigerant in the sequence was R-134a with a COP of 0.50 and an *f* of 5.7. Finally, HFO R-1234ze­(E) achieved a maximum COP of 0.44 with an *f* of 7.0. These differences were mostly due to the operating pressure of the absorber, which is dependent on the gas employed. The highest absorption pressure was 0.95 MPa for R-32, followed by the more condensable gases R-134a (0.35 MPa) and R-1234ze­(E) (0.26 MPa). These pressure differences resulted in greater absorption of R-32 in comparison to R-134a and R-1234ze­(E), which consequently led to enhanced operational performance of the ARS. This was achieved, however, at an elevated pressure, which in turn would lead to elevated process costs.

A comparison between the SE-ARS and CA-ARS results revealed that including a compression stage prior to absorption leads to enhanced COP and *f* values, enabling operation at reduced *T*_*g*_. In this instance, the maximum *T*_*g*_ values were approximately 20 K lower than those observed in the single stage cycle. In particular, at 328.15 K, the R-32/γ-valerolactone pair exhibited a COP of 0.71 (*f* = 4.7), the R-134a/γ-valerolactone pair a COP of 0.66 (*f* = 4), and the R-1234ze­(E)/γ-valerolactone pair a COP of 0.61 (*f* = 5). In addition, to assess the possibility of using the best CA-ARS designs over an expanded range of generator, absorber, and evaporator temperatures, a parametric evaluation of the CA-ARS performance was conducted, the results of which are shown in Section S11.

These findings represent a pioneering investigation into absorption pairs comprising low-GWP HFC/HFOs and green organic solvents. If the results of this work are compared with the existing literature on HFC-HFO/IL absorption pairs, several advances are evident. To illustrate these, Table 4 presents the best results from the literature using the same CA-ARS case study. In particular, Asensio-Delgado et al.^11^ reported the highest COP (0.73) for R-32, achieved at a *T*_*g*_ of 333.15 K with the IL [C_2_C_1_im]­[Tf_2_N]. In this study, almost equivalent COP values (0.70 and 0.71) were achieved using Rhodiasolv PolarClean and γ-valerolactone at a *T*_g_ 6 K lower, i.e., 327.15 K. The high solubility of R-134a in both γ-valerolactone and Rhodiasolv PolarClean resulted in absorption pairs with COP values nearly 20% higher than those observed with [C_2_C_1_im]­[Tf_2_N] and those reported by Wu et al.^13^ with the IL [C_6_C_1_im]­[Tf_2_N]. Additionally, γ-valerolactone exhibited a circulation factor almost half that of [C_2_C_1_im]­[Tf_2_N] and three times lower than that of [C_6_C_1_im]­[Tf_2_N].

**Table 4:** Comparison Between the CA-ARS Results Obtained with Green Organic Solvents and ILs for the Same Case Study

Working pair	T*_g_* (*K*)	COP	*f*	Reference
**R-32**				
γ-Valerolactone	327.15	0.71	4.7	This work
Rhodiasolv PolarClean	327.15	0.70	6.0	This work
[C_2_C_1_im][Tf_2_N]	333.15	0.73	5.4	^11^
[C_6_C_1_im][Tf_2_N]	328.15	0.67	6.1	^13^
**R-134a**				
γ-Valerolactone	328.15	0.66	4.0	This work
Rhodiasolv PolarClean	328.15	0.64	4.9	This work
[C_2_C_1_im][Tf_2_N]	333.15	0.62	7.2	^11^
[C_6_C_1_im][Tf_2_N]	328.15	0.53	11.6	^13^
**R-1234ze(E)**				
γ-valerolactone	328.15	0.61	5.0	This work
Rhodiasolv PolarClean	328.15	0.60	5.4	This work
[C_2_C_1_im][Tf_2_N]	333.15	0.38	22.4	^11^
[C_6_C_1_im][Tf_2_N]	333.15	0.43	17.4	^13^

Lastly, the most notable advance in this work lies in the performance of the absorption pairs involving HFO R-1234ze­(E). Notably, this refrigerant has an extremely low GWP, several orders of magnitude below that of HFCs R-32 and R-134a. Previous COP data for ARS using working fluids consisting of ILs and R-1234ze­(E) were low, with a maximum of 0.43 when R-1234ze­(E) was paired with [C_6_C_1_im]­[Tf_2_N].^13^ In contrast, the use of the green organic solvents investigated in this study significantly improved the COP, reaching 0.61 when R-1234ze­(E) was paired with γ-valerolactone and 0.60 with Rhodiasolv PolarClean. This represents an increase of nearly 50% in the COP value. At the same time, *f* decreased drastically to ratios of around 5, a significant advantage in terms of reducing the size of the ARS equipment and the cost of the solvent load. These results make R-1234ze­(E) competitive compared to working pairs based on high-GWP HFCs such as R-134a. Thus, for the first time, this work selects HFO/green organic solvent absorption pairs that not only offer a low environmental impact but also compete with HFC/IL absorption pairs.

As a final step in evaluating the viability of the proposed working pairs, their performance should be compared with the traditional pairs H_2_O/LiBr and NH_3_/H_2_O. In this regard, Sujatha and Venkatarathnam^14^ compared the SE-ARS performance of the IL [C_6_C_1_im]­[Tf_2_N] paired with R-32 and with the traditional working pairs at ARS conditions of *T*_a_/*T*_c_/*T*_g_/*T*_e_ 303.15/313.15/363.15/283.15 K. Their results, which included COP and *f* values, are compared in Table 5 with the CA-ARS results of the working pairs from this work. It is noteworthy that when a small compressor was installed before the absorption step, the biobased solvent working pairs achieved a higher COP than that of the traditional NH_3_/H_2_O system. Of particular significance was the case of R-1234ze­(E)/RPC, which matched the performance of NH_3_/H_2_O using an HFO with a very low GWP—a refrigerant that had not previously been considered a viable alternative. However, H_2_O/LiBr remained as the pair with the highest COP. Despite this advantage, H_2_O/LiBr presents notable shortcomings, such as salt crystallization issues and an inability to reduce the evaporator temperature, as it uses water as the refrigerant. Given the eco-friendly nature of the proposed pairs and their promising performance in simple ARS configurations, the results of this work clearly demonstrate their strong potential in this field. Future research should prioritize enhancing ARS efficiency with green organic solvents by optimizing system designs, improving solvent properties, and analyzing the effects of operational variables. Additionally, comprehensive economic and environmental assessments are necessary to ensure the long-term viability of ARS. These evaluations will be crucial in determining their feasibility as sustainable alternatives to conventional solvents in commercial applications.

**Table 5:** Comparison Between the Results Obtained with Green Organic Solvents and Traditional Working Pairs at *T*_a_/*T*_c_/*T*_g_/*T*_e_ 303.15/313.15/363.15/283.15 K^a^

Working pair	COP	f	ARS type	Reference
H_2_O/LiBr	0.83	4.47	Classical	^14^
NH_3_/H_2_O	0.56	2.58	Classical	^14^
R-32/GVL	0.65	2.16	CA-ARS	This work
R-32/RPC	0.64	2.61	CA-ARS	This work
R-134a/GVL	0.61	1.74	CA-ARS	This work
R-134a/RPC	0.54	1.98	CA-ARS	This work
R-1234ze(E)/GVL	0.49	2.08	CA-ARS	This work
R-1234ze(E)/RPC	0.55	2.18	CA-ARS	This work

a The compression ratio was 1.5.

## Conclusions and Future Work

This pioneering study analyzes the use of green organic solvents as fluorinated refrigerant gas absorbents in search of eco-friendly working fluids for refrigeration systems. The green organic solvents—solketal, propylene carbonate, terpinolene, γ-valerolactone, and Rhodiasolv PolarClean—are paired with both HFCs and HFOs. On the one hand, HFOs entail extremely low GWP factors, being the preferred option from the climate change mitigation perspective. On the other hand, HFCs could be the favored option if consistent circular economy approaches, based on gas collection and recycling, are introduced into the refrigeration market.

This work outlines a solvent selection pathway using physicochemical and environmental criteria. To enhance the sustainability of the ARS, the environmental, health, and safety impacts of the solvents are assessed by adjusting CHEM21 to the ideal solvent properties required for optimal performance. Moreover, their affinity and interactions with HFCs and HFOs are evaluated through quantum chemistry calculations, showing that most of the solvents exhibit a strong affinity for HFCs and HFOs, except for terpinolene, which presents limited compatibility with HFCs. The results indicate that while COSMO-RS is useful for qualitative screening, experimental validation is still needed due to its limited quantitative accuracy.

In a novel contribution to the field, we report experimental solubility data on HFCs and HFOs in green organic solvents. The high solubility of HFCs and HFOs in these solvents, combined with their suitable properties for refrigerant desorption, highlights their potential as effective absorbents to improve the sustainability of ARS. The NRTL model and the Peng–Robinson EoS, coupled with the Boston–Mathias mixing rule, are excellent methods for thermodynamically modeling these systems.

The performance of six absorption pairs comprising the refrigerants HFC-32, HFC-134a, and HFO-1234ze­(E), and the green solvents γ-valerolactone and Rhodiasolv PolarClean, in two ARS configurations, SE-ARS and CA-ARS, is evaluated. Among the refrigerants, the R-32 pairs show competitive results compared to those reported in the literature for other solvents. For R-134a and R-1234ze­(E), the results with these absorbents are greatly improved, leading to maximum COP values of 0.66 and 0.61, respectively. Notably, the R-1234ze­(E)-based pairs are the most promising, as they display the most favorable environmental profile due to the reduced GWP of the refrigerant. Furthermore, this is the first time in which a COP exceeding 0.6 has been achieved with this HFO. Overall, it can be concluded that green organic solvents hold significant potential as a valuable addition to ARS design, offering a competitive alternative to conventional working pairs while simultaneously improving the environmental profile.

## Supplementary Material



## Notes

Heat capacity measurements, experimental procedure for refrigerant solubilities, modeling approaches (NRTL and Peng–Robinson Boston–Mathias), procedure for modeling absorption refrigeration cycles, experimental VLE data, Henry’s law constants, enthalpy and entropy of solvation, and the parametric analysis on the ARS performance (PDF)
