# Supplementary material for: Biomass-Derived Solvents and Low-GWP Refrigerants as Working Fluids for Sustainable Absorption Refrigeration
Source: ACS Sustain Chem Eng. 2025 May 19;13(21):7728–39. doi: 10.1021/acssuschemeng.5c00258 (PMC12232040; doi:10.1021/acssuschemeng.5c00258)
Supplement: Supplementary file 1 [file sc5c00258_si_001.pdf]

SUPPORTING INFORMATION

Biomass-derived solvents and low-GWP refrigerants as working fluids for sustainable absorption refrigeration

Authors:

Miguel Viar, Fernando Pardo, Gabriel Zarca*, Ane Urtiaga*

Department of Chemical and Biomolecular Engineering, Universidad de Cantabria,

Av. Los Castros 46, 39005 Santander, Spain.

*Corresponding authors e-mail addresses:

Ane Urtiaga: urtiaga@unican.es

Gabriel Zarca: zarcag@unican.es

Supporting Information: 34 pages

Figures: 8

Tables: 23

CONTENTS

1. Heat capacity measurement 3

2. Experimental solubility measurement 4

3. Non-Random Two-Liquid (NRTL) model 7

4. Peng-Robinson Boston-Mathias 8

5. ARS modeling procedure 9

6. Experimental VLE data 12

7. Modeling results 19

8. Comparison between experimental solubility data and COSMO-RS 21

9. Henry law constants from experimental data and COSMO-RS 26

10. Enthalpy and entropy of solvation 29

11. Parametric analysis on the ARS performance 31

References 31

1. Heat capacity measurement

The heat capacity of Rhodiasolv® PolarClean was measured using two instruments: the C80 calorimeter, which was used to determine the heat capacity at four selected temperatures (298.15, 318.15, 338.15, and 358.15 K), and the MS80 calorimeter, which was used for determination/verification at 298.15 and 323.15 K.

Using the C80, the heat capacity was determined through a thermal scan performed under the same experimental conditions with an empty and a solvent-filled cell/container. The difference between the two signals was proportional to the thermal capacity of the solvent. The value obtained was then converted into heat energy using the instrument's impedance, which had been previously calibrated at each temperature. For the MS80, the operating principle was the same but performed in stages, obtaining energy values for the empty cell, a reference substance, and the solvent under study. In this work, decane and dodecane were selected as reference substances, with an uncertainty of ±1 J K^{-1} mol^{-1}.

The experimental heat capacity values of γ-valerolactone and Rhodiasolv® PolarClean are collected in Table S1.

Table S1. Experimental γ-valerolactone and Rhodiasolv® PolarClean heat capacity (C_p) at different temperatures.

**γ-Valerolactone** [1]			**Rhodiasolv® PolarClean (this work)**		
***T* (K)**	**C_p (J K^{-1} mol^{-1})**	**C_p (J K^{-1} g^{-1})**	***T* (K)**	**C_p (J K^{-1} mol^{-1})**	**C_p (J K^{-1} g^{-1})**
298.15	185	1.85	298.15	358	1.91
323.15	191	1.91	318.15	364	1.94
333.15	193	1.93	323.15	366	1.95
343.15	196	1.96	338.15	371	1.98
363.15	202	2.02	358.15	381	2.03

2. Experimental solubility measurement

The experimental set-up consisted on a jacketed stirred tank reactor (Buchi, Picoclave model, 170 mL), equipped with a pressure transducer (Keller, PAA-33X series, 0.01% accuracy) and a Pt-100 temperature sensor connected to a thermostatic bath (Grant, model LT ecocool 150, ±0.01 K). The reactor was connected to the storage cylinder (140 mL) by a valve, and it was equipped with another pressure transducer.

Approximately 30 g (±0.0001 g) of solvent was loaded into the absorption chamber, ensuring that the gas volume introduced was larger than the solvent to minimize the effect of its volumetric expansion along the gas absorption. Prior to each experiment, the solvent was heated at 333.15 K under vacuum conditions to remove any dissolved traces of water and volatile compounds. It is important to highlight that the solvent was never subjected to a pressure lower than its vapor pressure. Then, after adjusting the working temperature of the experiment, a certain amount of gas was introduced to the storage cylinder and the pressure and temperature were recorded. The connection valve was then opened, allowing for direct contact between the gas and solvent phases within the absorption chamber. The stirrer was set to a speed of 500 rpm to facilitate the absorption process, and the pressure and temperature were continuously recorded until equilibrium was achieved, that is, when the pressure remained constant for more than 20 min.

The solubility was calculated from the temperature and pressure measurements as the mole fraction of gas dissolved in the liquid phase:

$$x = \frac{n_{abs}}{n_l + n_{abs}} \tag{S1}$$

where n_{abs} are the total moles of gas dissolved and n_l are the moles of solvent loaded into the absorption chamber. Each isotherm consisted of various consecutive absorption steps, where the total gas absorbed in each step (n_i) was determined as follows:

$$n_i = \rho_{i,S} \cdot V_S + \rho_{i-1,C} \cdot (V_C - V_l) - \rho_{i,eq} \cdot (V_S + V_C - V_l) \tag{S2}$$

where V_S, V_C and V_l are the volumes of the storage cylinder, the absorption chamber, and the loaded solvent, and $\rho_{i,S}$, $\rho_{i-1,C}$ and $\rho_{i,eq}$ are the gas molar densities in the storage cylinder, in the absorption chamber, and at the equilibrium, respectively. From the experimental measurements of temperature and pressure, the molar densities were calculated by using the Peng-Robinson cubic equation of state (EoS) to account for deviations from the ideal behavior. However, given that the solvents under consideration in the present work do not exhibit a negligible vapor pressure, the pressure within the equilibrium cell was defined as follows [2–4]:

$$p_{eq} = p_C - p_i^0 \tag{S3}$$

where p_{eq}, p_C, and p_i^0 are the pressure of the refrigerant at the equilibrium, the pressure of the gas phase at the equilibrium in the absorption chamber, and the vapor pressure of the solvent used, respectively.

The total amount of gas dissolved was calculated as the amount absorbed in each step plus the amount dissolved in the previous stages (n_k):

$$n_{abs} = n_i + \sum_{k=1}^{i-1} n_k \tag{S4}$$

Once the solubility data were determined, the Henry's law constants (k_H) were calculated from:

$$k_H(T) = \lim_{x \to 0} \frac{\bar{f}(p,T)}{x} \tag{S5}$$

where $\bar{f}$ is the refrigerant gas fugacity calculated using the Peng-Robinson EoS. To determine the limit at infinite dilution, the experimental solubility was fitted to a second order polynomial [5,6].

The solubility uncertainty was calculated using the quadratic expansion of error, expanding each variable until considering all the measured properties (i.e., temperature, pressure, and mass). In particular, the uncertainty in molar faction was determined as follows:

$$u(x) = \sqrt{\left(\frac{\partial x}{\partial n_{abs}}\right)^2 \cdot \left(u(n_{abs})\right)^2 + \left(\frac{\partial x}{\partial n_l}\right)^2 \cdot \left(u(n_l)\right)^2} \tag{S6}$$

To validate the experimental set-up and procedure, the absorption isotherm of CO_2 and propylene carbonate (PC) was determined and compared with literature at 298.15 K. Table S2 records the experimental data, and Figure S1 represents both sets of data. Regarding the results, it could be concluded that the experimental methodology for the determination of VLE data was properly validated.

Table S2. VLE of CO_2 in PC at 298.15 K.

p (MPa)	x	$u(x)$
0.0583	0.0066	0.0002
0.1712	0.0195	0.0003
0.3136	0.0360	0.0005
0.5492	0.0630	0.0008
0.7297	0.0834	0.0010

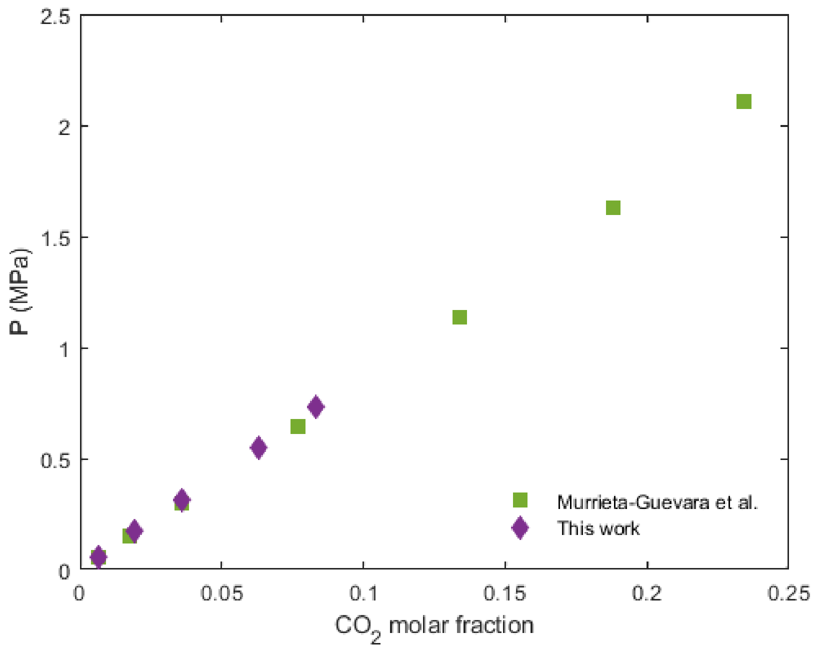

Figure S1. Absorption isotherm of CO_2 and PC at 298.15 K [7].

3. Non-Random Two-Liquid (NRTL) model

The NRTL activity coefficient model has been extensively utilized in previous research within this research field [8–10]. The VLE of each mixture component can be expressed as follows:

$$y_i \cdot p \cdot \Phi_i = x_i \cdot \gamma_i \cdot p_i^0 \quad (i \in \mathbb{Z}\,[1, N]) \tag{S7}$$

where x_i and y_i are the molar fraction of the compound i in the liquid and gas phase respectively, γ_i is the activity coefficient, and Φ_i is the Poynting correction factor. The latter is calculated as:

$$\Phi_i = \exp\left[\frac{\left(B_i - V_i^L\right) \cdot \left(p - p_i^0\right)}{R \cdot T}\right] \tag{S8}$$

where B_i is the second virial coefficient, V_i^L is the saturated liquid molar volume, T is the temperature of the system, and R is the ideal gas constant. The parameters B_i, V_i^L, and p_i^0 were estimated using the CoolProp 6.4.0 software, which implements multiparameter Helmholtz-energy-based equations of state that are specifically developed for each refrigerant [11].

For a binary mixture, the NRTL activity coefficient was calculated as follows:

$$\ln(\gamma_i) = x_2^2 \cdot \left[\tau_{21} \cdot \left(\frac{G_{21}}{x_1 + x_2 \cdot G_{21}}\right)^2 + \frac{\tau_{12} \cdot G_{12}}{(x_2 + x_1 \cdot G_{12})^2}\right] \tag{S9}$$

where

$$G_{12} = \exp(-\alpha \cdot \tau_{12}), \qquad G_{21} = \exp(-\alpha \cdot \tau_{21}) \tag{S10}$$

$$\tau_{12} = \tau_{12}^{(0)} + \frac{\tau_{12}^{(1)}}{T}, \qquad \tau_{21} = \tau_{21}^{(0)} + \frac{\tau_{21}^{(1)}}{T} \tag{S11}$$

The adjustable parameter α was previously assumed to be constant at 0.2 for fluorocarbons in accordance with the literature [12,13]. Subsequently, only the temperature-dependent binary interaction parameters τ_{12} and τ_{21} were optimized in this work as a function of two coefficients: $\tau_{12}^{(1)}$ and $\tau_{21}^{(1)}$ represent the excess Gibbs free energy divided by the ideal gas constant, while, $\tau_{12}^{(0)}$ and $\tau_{21}^{(0)}$ lack physical meaning, serving only to model large deviations from ideal behavior. The NRTL activity coefficients (γ_{calc}) were then fitted to the experimental values (γ_{exp}) by optimizing the binary interaction parameters in order to obtain the minimum average absolute relative deviation in activity coefficients ($AARD_\gamma$):

$$AARD_\gamma = \frac{100}{N}\sum_{i=1}^{N}\left|\frac{\gamma_{exp} - \gamma_{calc}}{\gamma_{exp}}\right| \tag{S12}$$

4. Peng-Robinson Boston-Mathias

The Peng-Robinson EoS coupled with the Boston-Mathias (PR-BM) mixing rule was also employed to regress the VLE data. The aforementioned mixing rule was applied in the EoS attractive parameter (a_m), as detailed below [14–16].

$$P = \frac{R \cdot T}{V_m - b_m} - \frac{a_m}{V_m \cdot (V_m + b_m) + b_m \cdot (V_m - b_m)} \tag{S13}$$

where V_m is the molar volume, a_m is the mixture attractive parameter, and b_m is the mixture covolume parameter. The two latter were calculated according to equation S14 and S15.

$$a_m = \sum_{i=1}^{N} \sum_{j=1}^{N} x_i x_j \sqrt{a_i a_j} \left(1 - k_{ij}\right) + \sum_{i=1}^{N} x_i \left(\sum_{j=1}^{N} x_j \left(\sqrt{a_i a_j} \cdot l_{ij}\right)^{\frac{1}{3}} \right)^3 \tag{S14}$$

$$b_m = \sum_{i=1}^{N} x_i \cdot b_i \tag{S15}$$

where

$$a_i = a_{c,i} \left(1 + \kappa_i \left(1 - \sqrt{T_{r,i}}\right)\right)^2 \tag{S16}$$

$$a_{c,i} = \frac{0.45724 \cdot R^2 \cdot T_{c,i}^2}{P_{c,i}} \tag{S17}$$

$$\kappa_i = 0.37464 + 1.54226 \cdot \omega_i - 0.2699 \omega_i^2 \tag{S18}$$

$$b_i = \frac{0.07780 \cdot R \cdot T_{c,i}}{P_{c,i}} \tag{S19}$$

where T_r and T_c are the reduced and critical temperature respectively, P_c is the critical pressure, and ω is the acentric factor. The binary interaction parameters, k_{ij} and l_{ij}, were defined as:

$$k_{ij} = k_{ij}^{(1)} + k_{ij}^{(2)} \cdot T + \frac{k_{ij}^{(3)}}{T}, \qquad k_{ij} = k_{ji} \tag{S20}$$

$$l_{ij} = l_{ij}^{(1)} + l_{ij}^{(2)} \cdot T + \frac{l_{ij}^{(3)}}{T}, \qquad l_{ij} \neq l_{ji} \tag{S21}$$

In order to regress the experimental data to the model, these parameters were modeled using Aspen Plus software (V12) with the maximum likelihood objective function.

5. ARS modeling procedure

Two absorption refrigeration systems, shown in Figure S2, were modeled with Matlab: the single-effect (SE-ARS) and the compression-assisted hybrid (CA-ARS). For this purpose, the following assumptions were made [17,18]:

1. The system operates in a steady-state condition.

2. The outlet solutions from the absorber and generator are in equilibrium.

3. Heat losses, flow resistance, and pressure drops are not considered.

4. The expansion valves produce isenthalpic pressure decrements.

5. Both the liquid and vapor phases are in saturated conditions.

6. The efficiency of the solution heat exchanger is set at 0.8.

7. Following the ASHRAE consideration, the outlet stream temperature of the generator is equal to the temperature of the inlet stream.

8. In the case of CA-ARS, the compressor isentropic efficiency (η_{comp}) and compression ratio (CR) are set at 0.7 and 1.5, respectively [17].

9. The pump operates isoenthalpically.

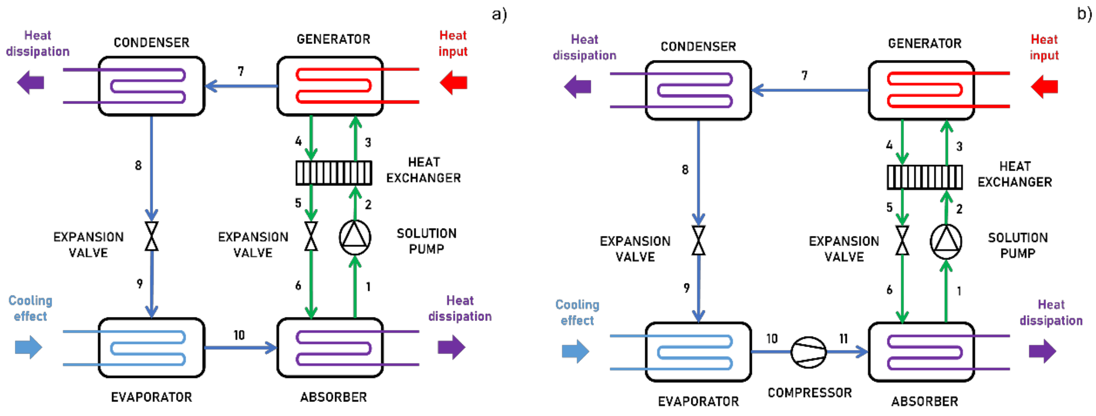

Figure S2. Schematic diagram of: a) SE-ARS, and b) CA-ARS.

The absorption cycles were modeled based on the following mass and energy balances. For the generator, the heat balance is:

$$Q_g + m_0 \cdot f \cdot h_3 = m_0 \cdot h_7 + m \cdot (f - 1) \cdot h_4 \quad \text{(S22)}$$

where Q_g is the generator heat exchange rate, m_0 is the mass flow rate of the refrigerant, f is the circulation factor, and h_i is the specific enthalpy of each stream. The circulation factor is

defined as the ratio between the solution mass flow leaving the absorber (m_{dil}) and the refrigerant mass flow used in the evaporator (m_r). This parameter is calculated from the mass balance to the refrigerant:

$$f = \frac{m_{dil}}{m_r} = \frac{1 - w_{con}}{\Delta w} = \frac{1 - w_{con}}{w_{dil} - w_{con}} \tag{S23}$$

where w_{dil} and w_{con} are the refrigerant mass fraction in the absorber and generator, respectively. The mass flow of the refrigerant (m_r) was fixed at 1 kg·s^{-1}.

For the absorber, condenser, evaporator, and the heat exchanger, the heat balances are described as follows respectively:

$$Q_a + m_0 \cdot f \cdot h_1 = m_0 \cdot (f - 1) \cdot h_6 + m_0 \cdot h_{10} \tag{S24}$$

$$Q_c + m_0 \cdot h_8 = m_0 \cdot h_7 \tag{S25}$$

$$Q_e + m_0 \cdot h_9 = m_0 \cdot h_{10} \tag{S26}$$

$$Q_x = m_0 \cdot f \cdot (h_3 - h_2) = m_0 \cdot (f - 1) \cdot (h_4 - h_5) \tag{S27}$$

Where Q_a, Q_c, Q_e, and Q_x are the heat exchange rate of the absorber, condenser, evaporator and heat exchanger, respectively. The state points parameters of the heat exchanger can be determined by:

$$\eta_x = \frac{T_4 - T_5}{T_4 - T_2} \tag{S28}$$

where η_x is the heat exchanger efficiency, set at 0.8 in this work [17]. For the solution pump, the heat balance is described as:

$$h_2 = h_1 + \frac{W_p}{m_0 \cdot f} \tag{S29}$$

Where W_p is the pump work, which is usually neglected due to its small percentage compared to the cooling capacity [17]. The pressure of the inlet stream to the absorber (p_a) in the CA-ARS is calculated as the product of the CR and the evaporator pressure (p_e).

$$p_a = p_e \cdot CR \tag{S30}$$

In addition to using the f as a measure of the ARS performance, the Coefficient of Performance (COP) is often used as a parameter to evaluate the efficiency of the system:

$$COP = \frac{Q_e}{Q_g} \tag{S31}$$

In the case of CA-ARS, COP is defined as:

$$COP = \frac{Q_e}{Q_g + W_{comp}} \tag{S32}$$

where W_{comp} is the energy of the compressor, calculated as follows:

$$W_{comp} = \frac{m_0 \cdot (h_{11} - h_{10})}{\eta_{comp}} \tag{S33}$$

The thermodynamic properties of the refrigerant gases were obtained using CoolProp 6.4.0 software [11]. The specific enthalpy of the solution of each stream was determined from:

$$h_i = x_{i_1} \cdot h_{i_1} + x_{i_2} \cdot h_{i_2} + h_{i_{ex}} \tag{S34}$$

where x_{i_1} and x_{i_2} are the molar fraction of the refrigerant and the green solvent, and $h_{i_{ex}}$ is the excess enthalpy of the stream, calculated from the NRTL parameters:

$$h_{ex} = -R \cdot T^2 \cdot \left[x_1 \cdot \left(\frac{\partial ln\gamma_1}{\partial T}\right)_{p,x} + x_2 \cdot \left(\frac{\partial ln\gamma_2}{\partial T}\right)_{p,x}\right] \tag{S35}$$

The enthalpies of the green solvents were obtained from their heat capacities, which were fitted to the following equation from experimental data:

$$Cp_i = Cp_0 \cdot T + Cp_1 \cdot \frac{T^2}{2} + Cp_2 \cdot \frac{T^3}{3} \tag{S36}$$

6. Experimental VLE data

Tables A2-A26 records the experimental VLE data of R-32, R-125, R-134a, R-1234yf, and R-1234ze(E) in SKL, PC, TPN, GVL, and RPC. The standard uncertainties are u(T)=0.01 K, and u(p)=0.001 bar. The standard uncertainties for molar fraction, u(x), are presented in the tables.

Table S3. Mole fraction solubility of R-32 in SKL and PC.

	R-32 + SKL			R-32 + PC		
T (K)	p (MPa)	x	$u(x)$	p (MPa)	x	$u(x)$
283.15	0.0397	0.0199	0.0002	0.0335	0.0186	0.0002
	0.1732	0.0886	0.0007	0.1455	0.0828	0.0006
	0.3608	0.1852	0.0011	0.3035	0.1785	0.0009
	0.5127	0.2650	0.0012	0.4280	0.2607	0.0011
	0.6362	0.3343	0.0014	0.5326	0.3366	0.0012
293.15	0.0450	0.0165	0.0002	0.0392	0.0163	0.0002
	0.1943	0.0738	0.0007	0.1703	0.0734	0.0006
	0.4061	0.1530	0.0010	0.3546	0.1572	0.0009
	0.5824	0.2200	0.0012	0.4980	0.2276	0.0011
	0.7201	0.2750	0.0015	0.6161	0.2891	0.0013
303.15	0.0483	0.0148	0.0002	0.0420	0.0134	0.0002
	0.2141	0.0636	0.0007	0.1878	0.0629	0.0006
	0.4445	0.1288	0.0010	0.3953	0.1354	0.0009
	0.6242	0.1806	0.0012	0.5585	0.1954	0.0011
	0.7808	0.2257	0.0016	0.6944	0.2480	0.0013
313.15	0.0501	0.0126	0.0002	0.0455	0.0121	0.0002
	0.2386	0.0501	0.0007	0.2030	0.0545	0.0006
	0.4894	0.1091	0.0011	0.4207	0.1131	0.0009
	0.6756	0.1533	0.0013	0.6066	0.1655	0.0011
	0.8292	0.1894	0.0017	0.7605	0.2106	0.0014
323.15	0.0635	0.0084	0.0002	0.0494	0.0102	0.0002
	0.2519	0.0432	0.0007	0.2280	0.0475	0.0006
	0.5186	0.0893	0.0011	0.4712	0.0987	0.0009
	0.7162	0.1231	0.0013	0.6614	0.1394	0.0011
	0.8752	0.1501	0.0017	0.8146	0.1746	0.0014

Table S4. Mole-fraction solubility of R-32 in GVL and RPC.

	R-32 + GVL			R-32 + RPC		
T (K)	p (MPa)	x	$u(x)$	p (MPa)	x	$u(x)$
283.15	0.0260	0.0208	0.0002	0.0230	0.0315	0.0003
	0.1145	0.0919	0.0006	0.1101	0.1379	0.0008
	0.2423	0.1935	0.0009	0.2404	0.2711	0.0011
	0.3521	0.2814	0.0010	0.3551	0.3710	0.0012
	0.4402	0.3530	0.0012	0.4553	0.4505	0.0013
	0.5369	0.4320	0.0013	0.5584	0.5276	0.0014
293.15	0.0311	0.0191	0.0002	0.0287	0.0293	0.0003
	0.1361	0.0831	0.0006	0.1273	0.1194	0.0008
	0.2919	0.1761	0.0009	0.2879	0.2482	0.0011
	0.4213	0.2536	0.0010	0.4258	0.3435	0.0012
	0.5322	0.3202	0.0012	0.5463	0.4172	0.0014
	0.6487	0.3888	0.0014	0.6619	0.4847	0.0015
303.15	0.0338	0.0164	0.0002	0.0344	0.0263	0.0003
	0.1535	0.0739	0.0006	0.1571	0.1155	0.0008
	0.3313	0.1576	0.0009	0.3401	0.2300	0.0012
	0.4787	0.2271	0.0010	0.4965	0.3155	0.0013
	0.6045	0.2863	0.0012	0.6285	0.3815	0.0014
	0.7394	0.3494	0.0015	0.7722	0.4472	0.0016
313.15	0.0385	0.0148	0.0002	0.0380	0.0230	0.0003
	0.1734	0.0663	0.0006	0.1744	0.1015	0.0008
	0.3731	0.1404	0.0009	0.3778	0.2032	0.0011
	0.5387	0.2011	0.0010	0.5473	0.2785	0.0013
	0.6790	0.2522	0.0013	0.6999	0.3381	0.0015
	0.8303	0.3067	0.0016	0.8623	0.3989	0.0018
323.15	0.0436	0.0134	0.0002	0.0457	0.0223	0.0003
	0.1927	0.0585	0.0006	0.1931	0.0825	0.0007
	0.4182	0.1247	0.0009	0.4133	0.1725	0.0011
	0.5907	0.1748	0.0010	0.607	0.2436	0.0013
	0.7413	0.2183	0.0013	0.7613	0.2952	0.0016
	0.8979	0.2603	0.0017	0.9321	0.3480	0.0019

Table S5. Mole-fraction solubility of R-32 and R-125 in TPN.

	R-32 + TPN			R-125 + TPN		
T (K)	p (MPa)	x	$u(x)$	p (MPa)	x	$u(x)$
283.15	0.0554	0.0112	0.0002	0.0578	0.0099	0.0002
	0.2362	0.0497	0.0006	0.1895	0.0335	0.0005
	0.4838	0.1090	0.0010	0.3231	0.0605	0.0007
	0.6617	0.1585	0.0012	0.4952	0.1003	0.0010
	0.7957	0.2028	0.0015	0.6553	0.1445	0.0013
293.15	0.0572	0.0094	0.0002	0.0606	0.0080	0.0002
	0.2500	0.0418	0.0006	0.1816	0.0249	0.0004
	0.5166	0.0911	0.0010	0.3306	0.0473	0.0006
	0.7024	0.1293	0.0012	0.5211	0.0793	0.0010
	0.8538	0.1641	0.0016	0.6737	0.1144	0.0012
303.15	0.0590	0.0085	0.0002	0.0647	0.0071	0.0002
	0.2604	0.0373	0.0006	0.1882	0.0207	0.0004
	0.5329	0.0801	0.0010	0.3421	0.0389	0.0006
	0.7228	0.1111	0.0012	0.5534	0.0657	0.0010
	0.8793	0.1385	0.0016	0.7137	0.0862	0.0013
313.15	0.0630	0.0069	0.0002	0.0644	0.0058	0.0002
	0.2691	0.0300	0.0006	0.1912	0.0168	0.0004
	0.5475	0.0623	0.0010	0.3534	0.0312	0.0006
	0.7468	0.0870	0.0012	0.5562	0.0491	0.0010
	0.9030	0.1075	0.0016	0.7318	0.0660	0.0013
323.15	0.0650	0.0065	0.0002	0.0649	0.0048	0.0002
	0.2756	0.0249	0.0006	0.1991	0.0142	0.0004
	0.5707	0.0515	0.0010	0.3598	0.0265	0.0006
	0.7712	0.0705	0.0012	0.5632	0.0416	0.0009
	0.9229	0.0855	0.0017	0.7335	0.0540	0.0013

Table S6. Mole-fraction solubility of R-125 in SKL and PC.

	R-125 + SKL			R-125 + PC		
T (K)	p (MPa)	x	$u(x)$	p (MPa)	x	$u(x)$
283.15	0.0479	0.0149	0.0002	0.0467	0.0115	0.0002
	0.1385	0.0564	0.0005	0.1409	0.0395	0.0005
	0.2434	0.1135	0.0007	0.2497	0.0824	0.0008
	0.3662	0.1946	0.0010	0.3621	0.1541	0.0010
	0.4709	0.2769	0.0012	0.4484	0.2377	0.0012
293.15	0.0545	0.0140	0.0002	0.0540	0.0101	0.0002
	0.1502	0.0453	0.0005	0.1590	0.0331	0.0005
	0.2713	0.0910	0.0007	0.2816	0.0674	0.0007
	0.4132	0.1544	0.0010	0.4277	0.1256	0.0011
	0.5441	0.2237	0.0012	0.5394	0.1924	0.0013
303.15	0.0529	0.0109	0.0002	0.0531	0.0073	0.0002
	0.1652	0.0386	0.0005	0.1622	0.0250	0.0005
	0.2961	0.0749	0.0007	0.3013	0.0523	0.0007
	0.4630	0.1280	0.0010	0.4683	0.0954	0.0010
	0.6080	0.1814	0.0013	0.6043	0.1445	0.0013
313.15	0.0647	0.0085	0.0002	0.0576	0.0063	0.0002
	0.1807	0.0299	0.0005	0.1796	0.0213	0.0005
	0.3283	0.0599	0.0007	0.3194	0.0410	0.0007
	0.5012	0.0990	0.0010	0.5012	0.0729	0.0011
	0.6527	0.1367	0.0013	0.6502	0.1075	0.0013
323.15	0.0653	0.0056	0.0002	0.0635	0.0047	0.0002
	0.1900	0.0225	0.0005	0.2036	0.0161	0.0005
	0.3364	0.0436	0.0007	0.3454	0.0306	0.0007
	0.5303	0.0734	0.0010	0.5331	0.0530	0.0011
	0.6959	0.1005	0.0014	0.6950	0.0767	0.0014

Table S7. Mole-fraction solubility of R-125 in GVL and RPC.

	R-125 + GVL			R-125 + RPC		
T (K)	p (MPa)	x	$u(x)$	p (MPa)	x	$u(x)$
283.15	0.0333	0.0187	0.0002	0.0263	0.0365	0.0003
	0.0916	0.0608	0.0004	0.0725	0.1109	0.0006
	0.1548	0.1245	0.0006	0.1268	0.2100	0.0009
	0.2219	0.2151	0.0009	0.1910	0.3277	0.0011
	0.2788	0.3039	0.0010	0.2541	0.4297	0.0011
	0.3308	0.3842	0.0011	0.3151	0.5109	0.0012
293.15	0.0406	0.0171	0.0002	0.0378	0.0371	0.0003
	0.1096	0.0532	0.0004	0.1001	0.1091	0.0006
	0.1884	0.1081	0.0006	0.1854	0.2209	0.0010
	0.2756	0.1892	0.0008	0.2738	0.3366	0.0011
	0.3490	0.2707	0.0010	0.3482	0.4232	0.0012
	0.4152	0.3475	0.0012	0.4205	0.4938	0.0013
303.15	0.0415	0.0135	0.0002	0.0373	0.0264	0.0003
	0.1250	0.0457	0.0004	0.1082	0.0861	0.0006
	0.2286	0.0979	0.0006	0.1906	0.1636	0.0008
	0.3355	0.1688	0.0008	0.2948	0.2670	0.0011
	0.4286	0.2436	0.0010	0.3924	0.3600	0.0012
	0.5111	0.3150	0.0012	0.4814	0.4358	0.0013
313.15	0.0610	0.0157	0.0002	0.0455	0.0261	0.0003
	0.1534	0.0433	0.0004	0.1220	0.0741	0.0005
	0.2581	0.0819	0.0006	0.2229	0.1439	0.0008
	0.3850	0.1414	0.0008	0.3490	0.2369	0.0011
	0.4990	0.2068	0.0010	0.4681	0.3241	0.0012
	0.5923	0.2666	0.0012	0.5843	0.4021	0.0014
323.15	0.0597	0.0119	0.0002	0.0441	0.0194	0.0003
	0.1582	0.0343	0.0004	0.1337	0.0619	0.0005
	0.2820	0.0665	0.0005	0.2468	0.1205	0.0008
	0.4316	0.1175	0.0008	0.3913	0.2009	0.0011
	0.5580	0.1696	0.0010	0.5216	0.2747	0.0013
	0.6654	0.2196	0.0013	0.6542	0.3471	0.0015

Table S8. Mole fraction solubility of R-134a in SKL and PC.

	R-134a + SKL			R-134a + PC		
T (K)	p (MPa)	x	$u(x)$	p (MPa)	x	$u(x)$
283.15	0.0384	0.0199	0.0002	0.0273	0.0214	0.0002
	0.0765	0.0574	0.0004	0.0650	0.0547	0.0003
	0.1221	0.1053	0.0006	0.1066	0.0987	0.0005
	0.1694	0.1594	0.0007	0.1478	0.1515	0.0007
	0.2194	0.2204	0.0009	0.1865	0.2128	0.0009
293.15	0.0401	0.0198	0.0002	0.0366	0.0185	0.0002
	0.0869	0.0529	0.0004	0.0821	0.0477	0.0003
	0.1429	0.0948	0.0006	0.1349	0.0869	0.0005
	0.2033	0.1433	0.0007	0.1862	0.1318	0.0007
	0.2658	0.1955	0.0010	0.2363	0.1841	0.0009
303.15	0.0431	0.0183	0.0002	0.0375	0.0161	0.0002
	0.0978	0.0465	0.0004	0.0917	0.0419	0.0003
	0.1613	0.0809	0.0005	0.1510	0.0738	0.0005
	0.2306	0.1208	0.0007	0.2135	0.1123	0.0006
	0.3043	0.1649	0.0010	0.2762	0.1568	0.0008
313.15	0.0466	0.0172	0.0002	0.0430	0.0144	0.0002
	0.1070	0.0409	0.0004	0.0995	0.0348	0.0003
	0.1781	0.0696	0.0005	0.1665	0.0613	0.0004
	0.2559	0.1024	0.0007	0.2390	0.0933	0.0006
	0.3382	0.1376	0.0010	0.3126	0.1296	0.0008
323.15	0.0500	0.0139	0.0002	0.0460	0.0118	0.0002
	0.1184	0.0339	0.0004	0.1091	0.0292	0.0003
	0.1948	0.0569	0.0005	0.1828	0.0512	0.0004
	0.2806	0.0828	0.0007	0.2615	0.0766	0.0006
	0.3660	0.1074	0.0010	0.3443	0.1053	0.0008

Table S9. Mole-fraction solubility of R-134a in GVL and RPC.

	R-134a + GVL			R-134a + RPC		
T (K)	p (MPa)	x	$u(x)$	p (MPa)	x	$u(x)$
283.15	0.0176	0.0248	0.0002	0.0147	0.0406	0.0003
	0.0420	0.0631	0.0003	0.0403	0.1110	0.0006
	0.0706	0.1120	0.0005	0.0687	0.1869	0.0007
	0.1011	0.1681	0.0007	0.0990	0.2644	0.0009
	0.1337	0.2275	0.0008	0.1300	0.3397	0.0011
293.15	0.0224	0.0218	0.0002	0.0185	0.0367	0.0003
	0.0544	0.0571	0.0003	0.0461	0.0912	0.0005
	0.0913	0.1014	0.0005	0.0812	0.1585	0.0007
	0.1317	0.1528	0.0006	0.1202	0.2316	0.0009
	0.1754	0.2084	0.0008	0.1618	0.3061	0.0010
303.15	0.0265	0.0202	0.0002	0.0227	0.0329	0.0003
	0.0661	0.0527	0.0003	0.0580	0.0840	0.0005
	0.1114	0.0925	0.0005	0.1015	0.1458	0.0007
	0.1612	0.1375	0.0006	0.1496	0.2124	0.0009
	0.2218	0.1850	0.0008	0.2008	0.2805	0.0010
313.15	0.0312	0.0184	0.0002	0.0278	0.0301	0.0003
	0.0756	0.0458	0.0003	0.0714	0.0773	0.0005
	0.1298	0.0812	0.0004	0.1250	0.1344	0.0007
	0.1897	0.1207	0.0006	0.1840	0.1958	0.0009
	0.2576	0.1600	0.0008	0.2429	0.2555	0.0011
323.15	0.0352	0.0166	0.0002	0.0325	0.0266	0.0003
	0.0855	0.0417	0.0003	0.0849	0.0697	0.0005
	0.1446	0.0729	0.0004	0.1412	0.1157	0.0006
	0.2082	0.1084	0.0006	0.2075	0.1686	0.0008
	0.2804	0.1459	0.0008	0.2766	0.2223	0.0011

Table S10. Mole-fraction solubility of R-134a in TPN.

	R-134a + TPN		
T (K)	p (MPa)	x	$u(x)$
283.15	0.0461	0.0160	0.0002
	0.1083	0.0402	0.0004
	0.1776	0.0708	0.0005
	0.2429	0.1049	0.0007
	0.3058	0.1449	0.0009
293.15	0.0499	0.0139	0.0002
	0.1176	0.0341	0.0003
	0.1941	0.0585	0.0005
	0.2655	0.0849	0.0007
	0.3348	0.1136	0.0009
303.15	0.0550	0.0124	0.0002
	0.1286	0.0298	0.0003
	0.2072	0.0502	0.0005
	0.2819	0.0708	0.0007
	0.3591	0.0937	0.0009
313.15	0.0579	0.0106	0.0002
	0.1327	0.0243	0.0003
	0.2187	0.0408	0.0005
	0.3051	0.0582	0.0007
	0.3809	0.0744	0.0009
323.15	0.0598	0.0092	0.0002
	0.1352	0.0205	0.0003
	0.2236	0.0340	0.0005
	0.3052	0.0475	0.0006
	0.3835	0.0610	0.0009

Table S11. Mole-fraction solubility of R-1234yf in SKL and PC.

	R-1234yf + SKL			R-1234yf + PC		
T (K)	p (MPa)	x	$u(x)$	p (MPa)	x	$u(x)$
283.15	0.0419	0.0178	0.0002	0.0512	0.0105	0.0002
	0.0986	0.0450	0.0004	0.1110	0.0276	0.0003
	0.1628	0.0809	0.0005	0.1802	0.0510	0.0004
	0.2282	0.1261	0.0007	0.2491	0.0805	0.0006
	0.2874	0.1789	0.0009	0.3143	0.1186	0.0008
293.15	0.0478	0.0146	0.0002	0.0523	0.0089	0.0002
	0.1122	0.0364	0.0004	0.1197	0.0230	0.0003
	0.1839	0.0635	0.0005	0.1949	0.0410	0.0004
	0.2610	0.0976	0.0007	0.2729	0.0631	0.0005
	0.3359	0.1373	0.0009	0.3527	0.0907	0.0007
303.15	0.0513	0.0122	0.0002	0.0569	0.0074	0.0002
	0.1197	0.0302	0.0003	0.1349	0.0194	0.0003
	0.1978	0.0529	0.0005	0.2171	0.0343	0.0004
	0.2800	0.0792	0.0007	0.2983	0.0511	0.0005
	0.3630	0.1100	0.0009	0.3835	0.0710	0.0008
313.15	0.0551	0.0104	0.0002	0.0641	0.0065	0.0002
	0.1280	0.0251	0.0003	0.1356	0.0154	0.0003
	0.2104	0.0429	0.0005	0.2194	0.0266	0.0004
	0.2999	0.0640	0.0007	0.3107	0.0398	0.0005
	0.3872	0.0864	0.0009	0.4022	0.0542	0.0007
323.15	0.0588	0.0084	0.0002	0.0648	0.0048	0.0002
	0.1332	0.0201	0.0003	0.1439	0.0124	0.0003
	0.2190	0.0343	0.0005	0.2309	0.0212	0.0004
	0.3089	0.0498	0.0007	0.3185	0.0303	0.0005
	0.4026	0.0669	0.0009	0.4138	0.0407	0.0007

Table S12. Mole-fraction solubility of R-1234yf in GVL and RPC.

	R-1234yf + GVL			R-1234yf + RPC		
T (K)	p (MPa)	x	$u(x)$	p (MPa)	x	$u(x)$
283.15	0.0361	0.0161	0.0002	0.0326	0.0291	0.0003
	0.0855	0.0422	0.0003	0.0758	0.0714	0.0005
	0.1380	0.0772	0.0004	0.1256	0.1270	0.0006
	0.1873	0.1207	0.0006	0.1743	0.1913	0.0008
	0.2309	0.1733	0.0008	0.2195	0.2620	0.0010
293.15	0.0456	0.0157	0.0002	0.0374	0.0244	0.0003
	0.1016	0.0380	0.0003	0.0957	0.0669	0.0005
	0.1595	0.0659	0.0004	0.1557	0.1164	0.0006
	0.2199	0.1020	0.0006	0.2185	0.1760	0.0009
	0.2767	0.1459	0.0008	0.2734	0.2366	0.0011
303.15	0.0512	0.0137	0.0002	0.0435	0.0223	0.0003
	0.1135	0.0325	0.0003	0.1013	0.0541	0.0004
	0.1810	0.0562	0.0004	0.1668	0.0938	0.0006
	0.2501	0.0852	0.0006	0.2361	0.1401	0.0008
	0.3178	0.1201	0.0008	0.3170	0.1974	0.0011
313.15	0.0523	0.0110	0.0002	0.0500	0.0199	0.0011
	0.1188	0.0265	0.0003	0.1119	0.0462	0.0012
	0.1905	0.0452	0.0004	0.1858	0.0798	0.0015
	0.2687	0.0689	0.0005	0.2648	0.1187	0.0020
	0.3481	0.0970	0.0007	0.3432	0.1613	0.0025
323.15	0.0532	0.0089	0.0002	0.0519	0.0163	0.0002
	0.1233	0.0213	0.0003	0.1199	0.0389	0.0004
	0.2030	0.0372	0.0004	0.1956	0.0653	0.0006
	0.2886	0.0562	0.0005	0.2736	0.0955	0.0008
	0.3746	0.0781	0.0007	0.3639	0.1266	0.0010

Table S13. Mole fraction solubility of R-1234yf and R-1234ze(E) in TPN.

	R-1234yf + TPN			R-1234ze(E) + TPN		
T (K)	p (MPa)	x	$u(x)$	p (MPa)	x	$u(x)$
283.15	0.0409	0.0192	0.0002	0.0317	0.0239	0.0002
	0.0980	0.0491	0.0004	0.0742	0.0618	0.0004
	0.1592	0.0865	0.0005	0.1226	0.1102	0.0006
	0.2209	0.1328	0.0007	0.1621	0.1567	0.0007
	0.2767	0.1876	0.0009	0.1961	0.2061	0.0009
293.15	0.0453	0.0169	0.0002	0.0388	0.0202	0.0002
	0.1070	0.0419	0.0004	0.0900	0.0509	0.0004
	0.1763	0.0735	0.0005	0.1485	0.0913	0.0005
	0.2465	0.1111	0.0007	0.1967	0.1295	0.0007
	0.3170	0.1566	0.0009	0.2356	0.1637	0.0009
303.15	0.0511	0.0149	0.0002	0.0437	0.0187	0.0002
	0.1162	0.0354	0.0003	0.0997	0.0470	0.0004
	0.1907	0.0614	0.0005	0.1631	0.0822	0.0005
	0.2710	0.0927	0.0007	0.2163	0.1147	0.0007
	0.3517	0.1285	0.0009	0.2671	0.1483	0.0009
313.15	0.0537	0.0132	0.0002	0.0458	0.0164	0.0002
	0.1229	0.0307	0.0003	0.1114	0.0417	0.0004
	0.2101	0.0545	0.0005	0.1814	0.0706	0.0005
	0.2908	0.0788	0.0007	0.2390	0.0965	0.0007
	0.3769	0.1070	0.0009	0.2952	0.1227	0.0009
323.15	0.0573	0.0117	0.0002	0.0506	0.0153	0.0002
	0.1381	0.0283	0.0004	0.1152	0.0358	0.0003
	0.2187	0.0458	0.0005	0.1912	0.0615	0.0005
	0.3030	0.0654	0.0007	0.2530	0.0830	0.0007
	0.3922	0.0879	0.0009	0.3153	0.1038	0.0009

Table S14. Mole-fraction solubility of R-1234ze(E) in SKL and PC.

	R-1234ze(E) + SKL			R-1234ze(E) + PC		
T (K)	p (MPa)	x	$u(x)$	p (MPa)	x	$u(x)$
283.15	0.0254	0.0275	0.0002	0.0297	0.0186	0.0002
	0.0593	0.0690	0.0003	0.0656	0.0483	0.0003
	0.0986	0.1211	0.0006	0.1061	0.0877	0.0004
	0.1331	0.1703	0.0006	0.1400	0.1276	0.0006
	0.1654	0.2201	0.0009	0.1719	0.1702	0.0008
293.15	0.0327	0.0224	0.0002	0.0365	0.0147	0.0002
	0.0740	0.0576	0.0003	0.0803	0.0407	0.0003
	0.1245	0.1026	0.0005	0.1304	0.0746	0.0004
	0.1666	0.1449	0.0006	0.1727	0.1088	0.0006
	0.2071	0.1885	0.0009	0.2110	0.1443	0.0007
303.15	0.0385	0.0201	0.0002	0.0373	0.0142	0.0002
	0.0863	0.0510	0.0004	0.0882	0.0367	0.0003
	0.1432	0.0900	0.0005	0.1481	0.0662	0.0004
	0.1931	0.1253	0.0007	0.1987	0.0944	0.0005
	0.2406	0.1619	0.0009	0.2451	0.1240	0.0007
313.15	0.0422	0.0173	0.0002	0.0425	0.0123	0.0002
	0.0974	0.0437	0.0003	0.1008	0.0318	0.0003
	0.1610	0.0757	0.0005	0.1675	0.0559	0.0004
	0.2168	0.1048	0.0006	0.2240	0.0787	0.0005
	0.2702	0.1344	0.0009	0.2778	0.1022	0.0007
323.15	0.0455	0.0151	0.0002	0.0466	0.0113	0.0002
	0.1079	0.0366	0.0003	0.1089	0.0273	0.0003
	0.1799	0.0628	0.0005	0.1818	0.0474	0.0004
	0.2407	0.0857	0.0006	0.2417	0.0652	0.0005
	0.3005	0.1086	0.0008	0.3011	0.0833	0.0007

Table S15. Mole-fraction solubility of R-1234ze(E) in GVL and RPC.

	R-1234ze(E) + GVL			R-1234ze(E) + RPC		
T (K)	p (MPa)	x	$u(x)$	p (MPa)	x	$u(x)$
283.15	0.0180	0.0250	0.0002	0.0141	0.0401	0.0003
	0.0430	0.0644	0.0003	0.0381	0.1062	0.0005
	0.0713	0.1143	0.0005	0.0632	0.1760	0.0007
	0.0965	0.1628	0.0006	0.0872	0.2401	0.0009
	0.1209	0.2123	0.0008	0.1114	0.3033	0.0010
293.15	0.0237	0.0231	0.0002	0.0200	0.0368	0.0003
	0.0555	0.0585	0.0003	0.0464	0.0933	0.0005
	0.0926	0.1034	0.0005	0.0794	0.1600	0.0007
	0.1256	0.1475	0.0006	0.1103	0.2227	0.0008
	0.1574	0.1924	0.0008	0.1415	0.2826	0.0010
303.15	0.0288	0.0219	0.0002	0.0224	0.0344	0.0003
	0.0685	0.0546	0.0003	0.0549	0.0850	0.0005
	0.1126	0.0941	0.0005	0.0968	0.1484	0.0007
	0.1525	0.1325	0.0006	0.1336	0.2035	0.0008
	0.1913	0.1720	0.0008	0.1720	0.2576	0.0010
313.15	0.0324	0.0182	0.0002	0.0272	0.0303	0.0003
	0.0790	0.0456	0.0003	0.0693	0.0781	0.0006
	0.1333	0.0803	0.0004	0.1175	0.1315	0.0007
	0.1792	0.1134	0.0006	0.1621	0.1802	0.0008
	0.2239	0.1479	0.0008	0.2078	0.2280	0.0010
323.15	0.0375	0.0153	0.0002	0.0330	0.0285	0.0003
	0.0927	0.0402	0.0003	0.0796	0.0687	0.0004
	0.1518	0.0696	0.0004	0.1364	0.1171	0.0006
	0.2031	0.0972	0.0006	0.1880	0.1608	0.0008
	0.2556	0.1261	0.0008	0.2390	0.2026	0.0010

7. Modeling results

Tables S16 and S17 records the fitting results of the NRTL and PR-BM approaches to the experimental data.

Table S16. NRTL model parameters for each gas/solvent pair under study.

Gas	Solvent	α	$\tau_{12}^{(0)}$	$\tau_{12}^{(1)}$	$\tau_{21}^{(0)}$	$\tau_{21}^{(1)}$	$AARD_{\gamma}$
R-32	SKL	0.2	0	4938.5	0	70.593	3.08
	PC	0.2	0	6154.7	0	117.17	1.06
	TPN	0.2	0	922.70	0	-7.5614	2.59
	GVL	0.2	0	673.22	0	-332.67	1.86
	RPC	0.2	0	7518.4	0	-105.73	1.32
R-125	SKL	0.2	0.2407	8039.7	3.0529	-516.45	5.13
	PC	0.2	6.0995	10186	3.6840	-570.99	3.25
	TPN	0.2	0	4311.0	0	384.42	1.76
	GVL	0.2	0.5444	-519.91	1.7518	223.63	1.60
	RPC	0.2	0	-626.20	0	1035.2	5.42
R-134a	SKL	0.2	0	621.10	0	-37.989	4.67
	PC	0.2	0	6701.9	0	314.93	1.97
	TPN	0.2	0	800.17	0	124.73	1.38
	GVL	0.2	0	7145.7	0	144.31	2.20
	RPC	0.2	0	7670.8	0	-44.901	2.85
R-1234yf	SKL	0.2	0	4910.9	0	379.70	1.49
	PC	0.2	0	6091.2	0	637.71	3.92
	TPN	0.2	0	621.80	0	94.028	1.31
	GVL	0.2	0	6647.0	0	456.43	1.74
	RPC	0.2	0	6100.1	0	237.17	0.76
R-1234ze(E)	SKL	0.2	0	5211.7	0	222.31	2.33
	PC	0.2	0	6442.9	0	427.45	2.87
	TPN	0.2	0	629.97	0	47.469	2.36
	GVL	0.2	0	6197.3	0	198.80	1.87
	RPC	0.2	0	6927.6	0	17.883	1.44

Table S17. PR-BM model parameter for each refrigerant gas/green solvent under study.

**Gas/Solvent**	$k_{ij}^{(1)}$	$k_{ij}^{(2)} \cdot 10^3$	$k_{ij}^{(3)}$	$l_{ij}^{(1)}$	$l_{ij}^{(2)} \cdot 10^3$	$l_{ij}^{(3)}$	$AARD_x$
R-32/SKL	0.1133	-0.2253	0	0.7941	-2.5824	0	0.37
R-32/PC	-0.0319	0.0395	0	-0.0315	0.0488	0	0.14
R-32/TPN	-0.1484	1.0965	0	-0.2556	0.6899	0	0.50
R-32/GVL	-0.0146	0.0067	0	-0.0562	0.1992	0	0.12
R-32/RPC	-1.5012	2.4771	217.00	-0.1432	0.3186	21.425	0.11
R-125/SKL	0.1182	-0.6068	0	0.8904	-3.3950	0	0.45
R-125/PC	0.0543	-0.5925	0	0.5728	-2.4711	0	0.28
R-125/TPN	-0.1891	1.1349	0	-0.2156	0.4619	0	0.21
R-125/GVL	-0.7031	0.9174	92.554	0.2816	-0.9134	-43.850	0.04
R-125/RPC	-0.7957	1.1968	93.529	-3.1504	5.1521	449.59	0.11
R-134a/SKL	2.4807	-3.3386	0	16.232	-24.747	0	0.54
R-134a/PC	-0.0667	0.0415	0	-0.0980	0.1306	0	0.17
R-134a/TPN	-0.0571	0.7187	0	-0.2393	0.6119	0	0.15
R-134a/GVL	-0.0893	0.1645	0	-0.2118	0.6172	0	0.19
R-134a/RPC	-0.0371	-0.0170	0	0.0635	-0.2349	0	0.58
R-1234yf/SKL	-0.0341	0.1258	0	0.2412	-0.9196	0	0.15
R-1234yf/PC	0.2606	-1.1210	0	0.8082	-3.1653	0	0.30
R-1234yf/TPN	-0.0184	0.4041	0	-0.1461	0.2963	0	0.10
R-1234yf/GVL	0.0215	0.0720	0	0.0523	0.1747	0	0.09
R-1234yf/RPC	-0.0686	0.2071	0	-0.0493	-0.0081	0	0.09
R-1234ze(E)/SKL	-0.0541	0.1882	0	0.0887	-0.3194	0	0.21
R-1234ze(E)/PC	-0.1243	0.2483	0	-0.3160	0.8594	0	0.17
R-1234ze(E)/TPN	-0.0194	0.4225	0	-0.2839	0.7999	0	0.27
R-1234ze(E)/GVL	$-4.89 \cdot 10^{-4}$	-0.1055	0	0.1764	-0.6638	0	0.10
R-1234ze(E)/RPC	-0.0201	-0.0226	0	0.0086	-0.0279	0	0.15

8. Comparison between experimental solubility data and COSMO-RS

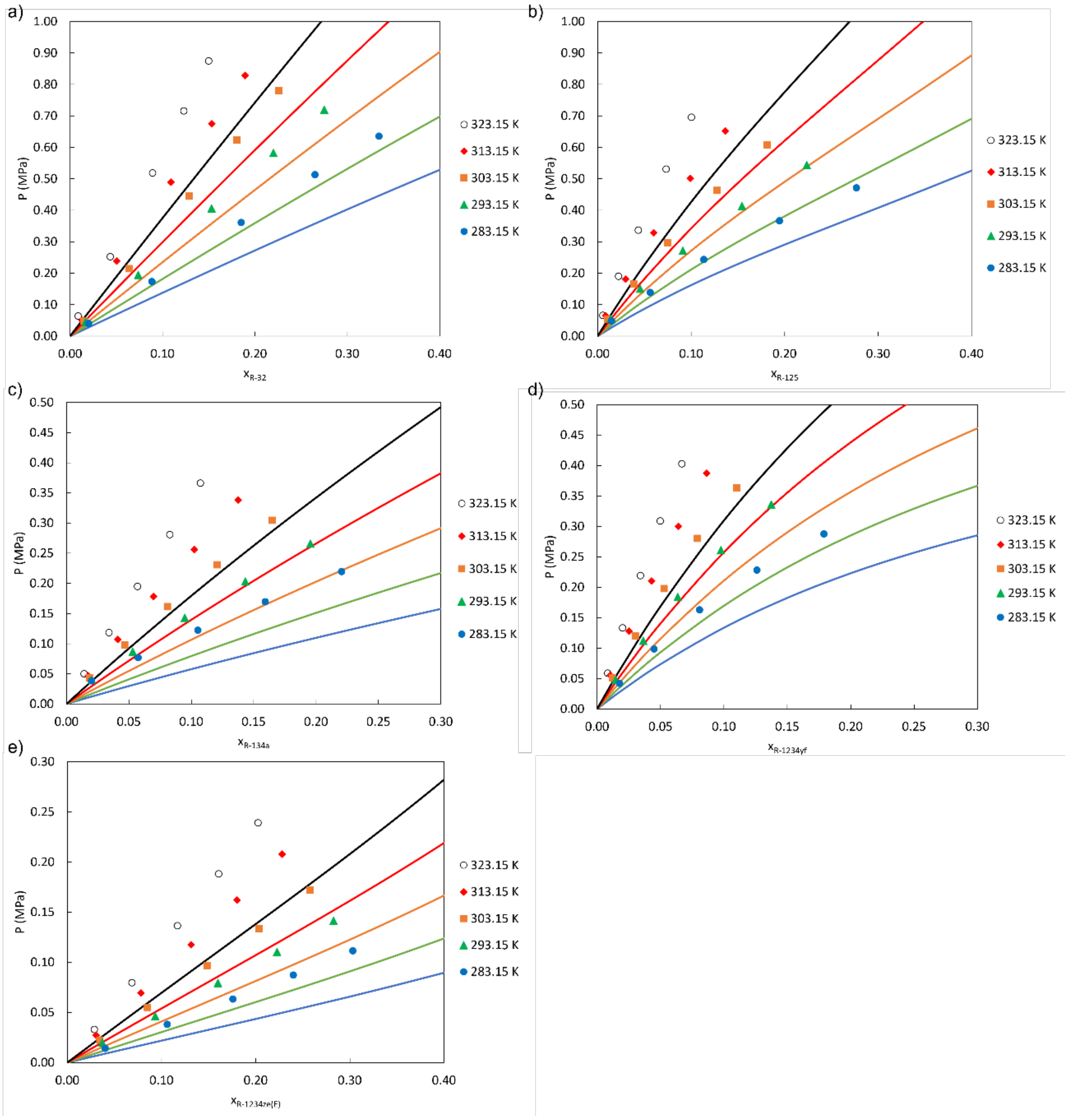

Figure S3. Solubility isotherms of: a) R-32, b) R-125, c) R-134a, d) R-1234yf, and e) R-1234ze(E), in SKL. The points are the experimental data, and lines represent the isotherms from COSMO-RS.

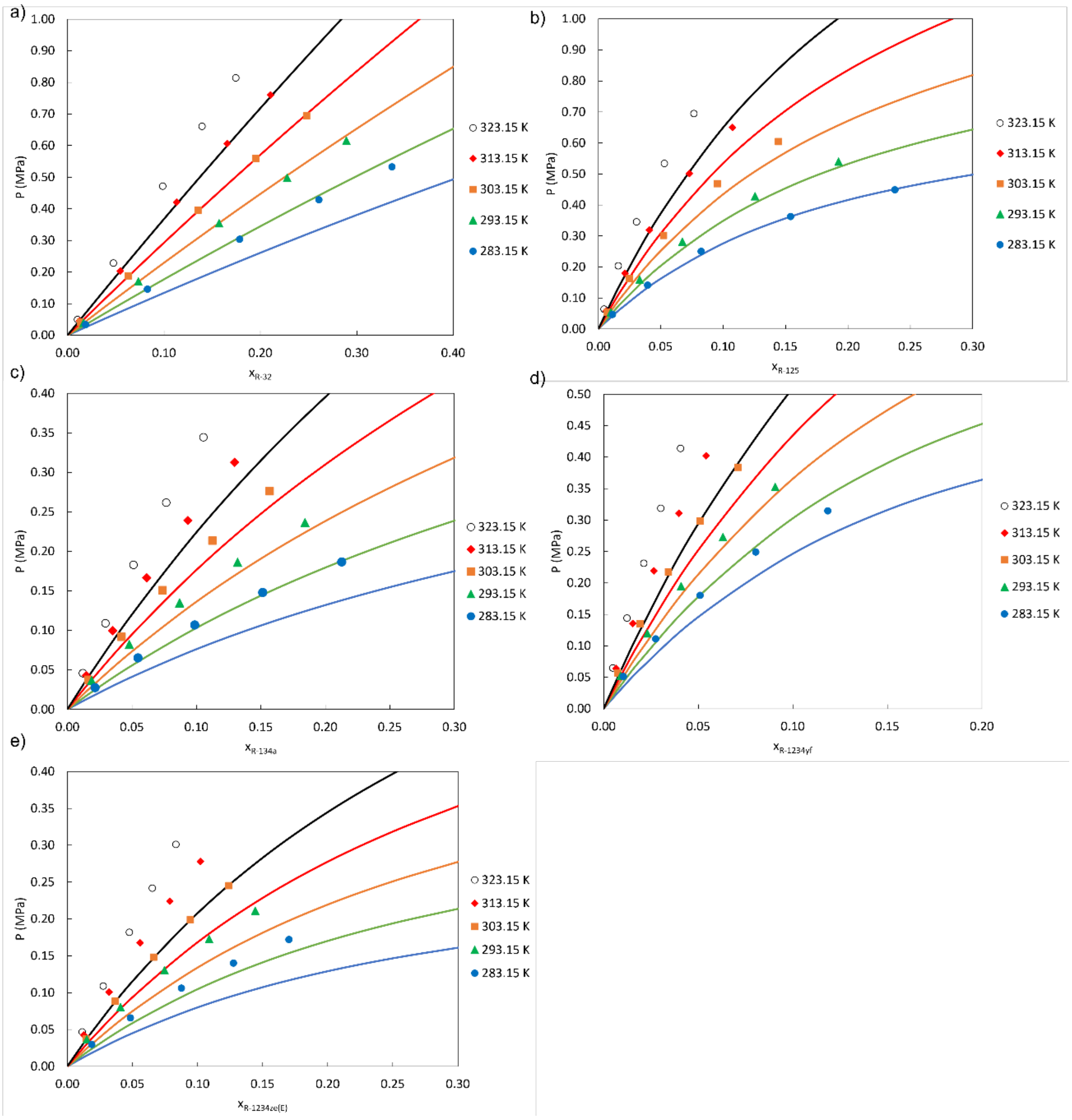

Figure S4. Solubility isotherms of: a) R-32, b) R-125, c) R-134a, d) R-1234yf, and e) R-1234ze(E), in PC. The points are the experimental data, and lines represent the isotherms from COSMO-RS.

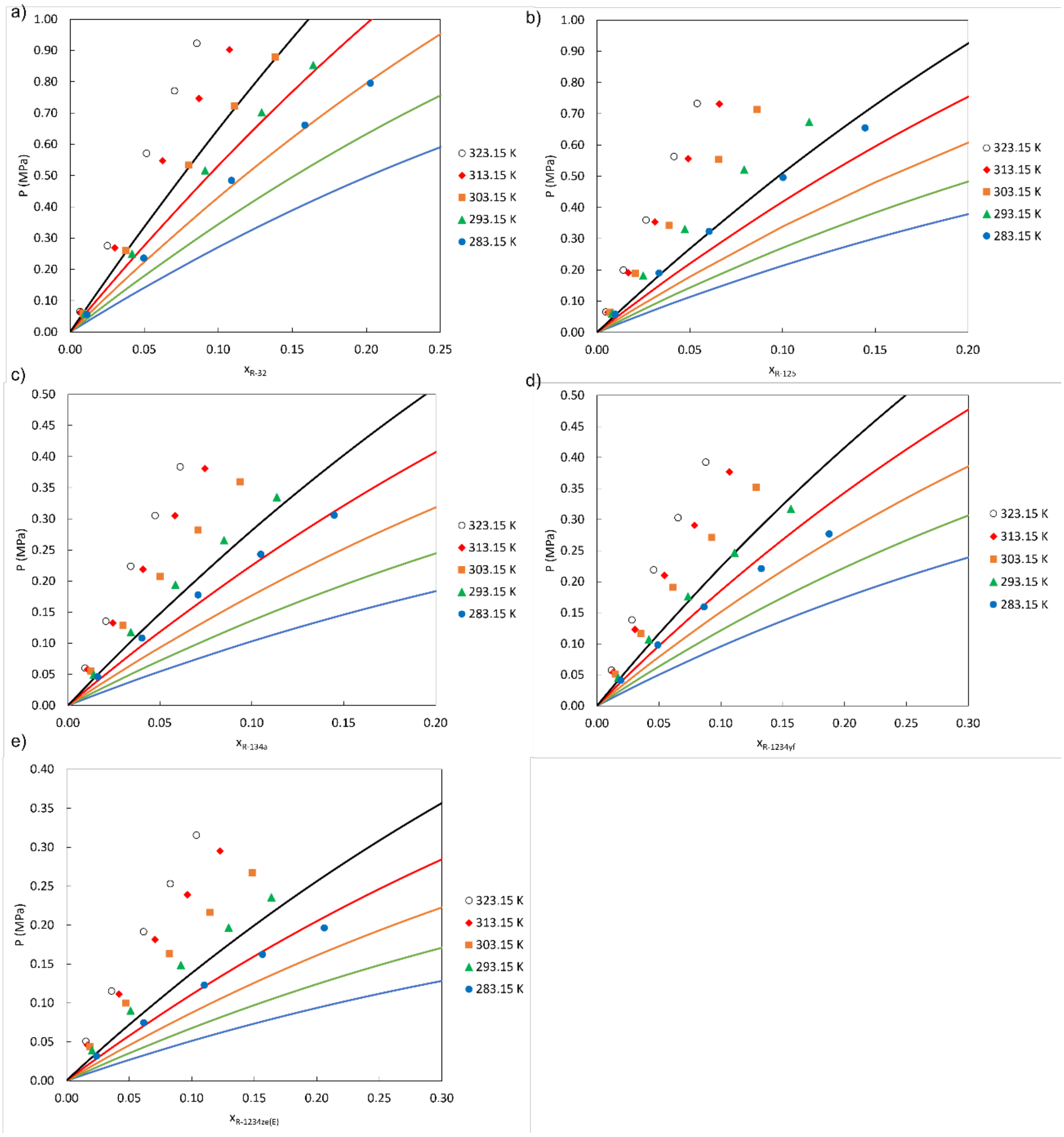

Figure S5. Solubility isotherms of: a) R-32, b) R-125, c) R-134a, d) R-1234yf, and e) R-1234ze(E), in TPN. The points are the experimental data, and lines represent the isotherms from COSMO-RS.

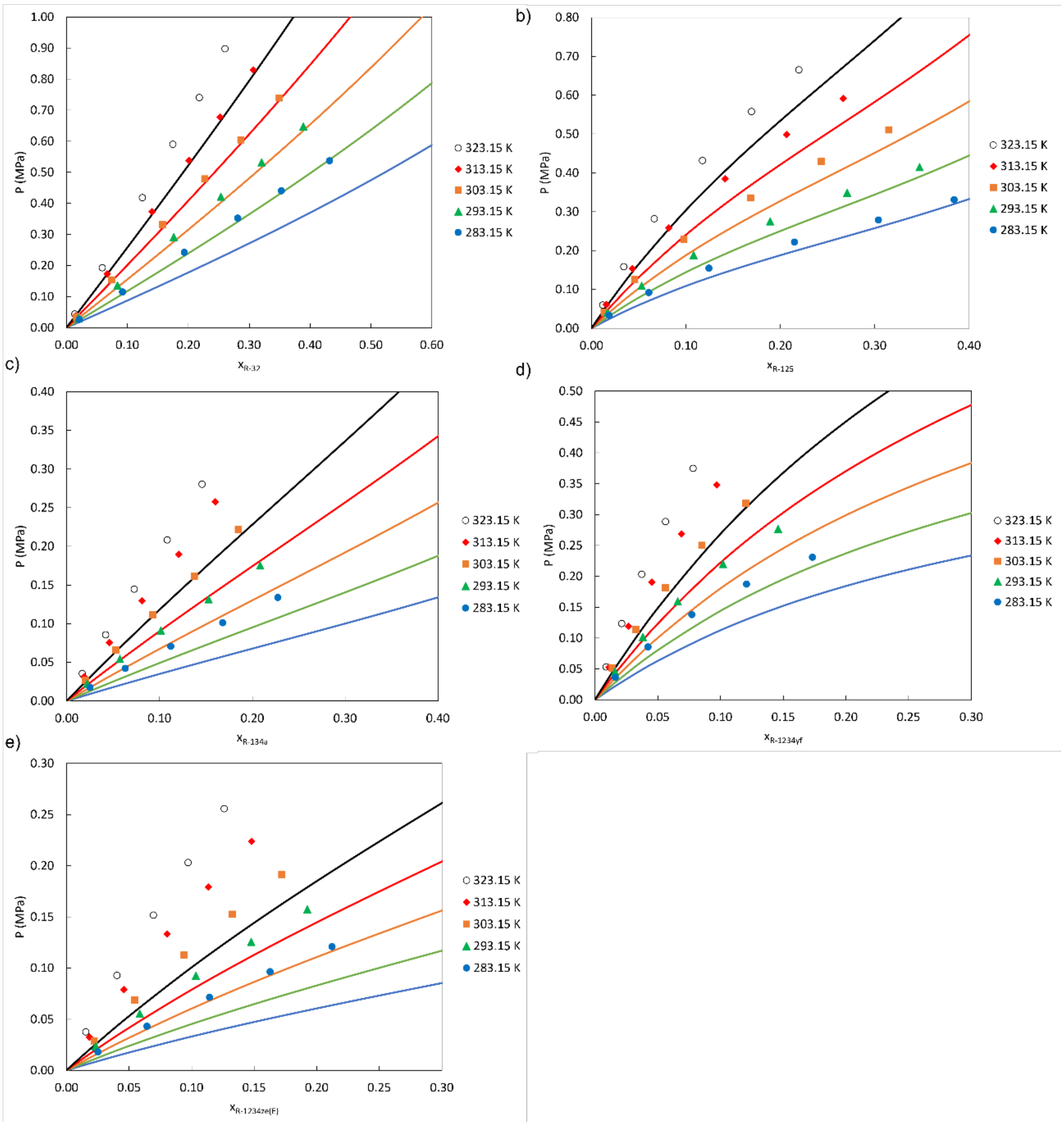

Figure S6. Solubility isotherms of: a) R-32, b) R-125, c) R-134a, d) R-1234yf, and e) R-1234ze(E), in GVL. The points are the experimental data, and lines represent the isotherms from COSMO-RS.

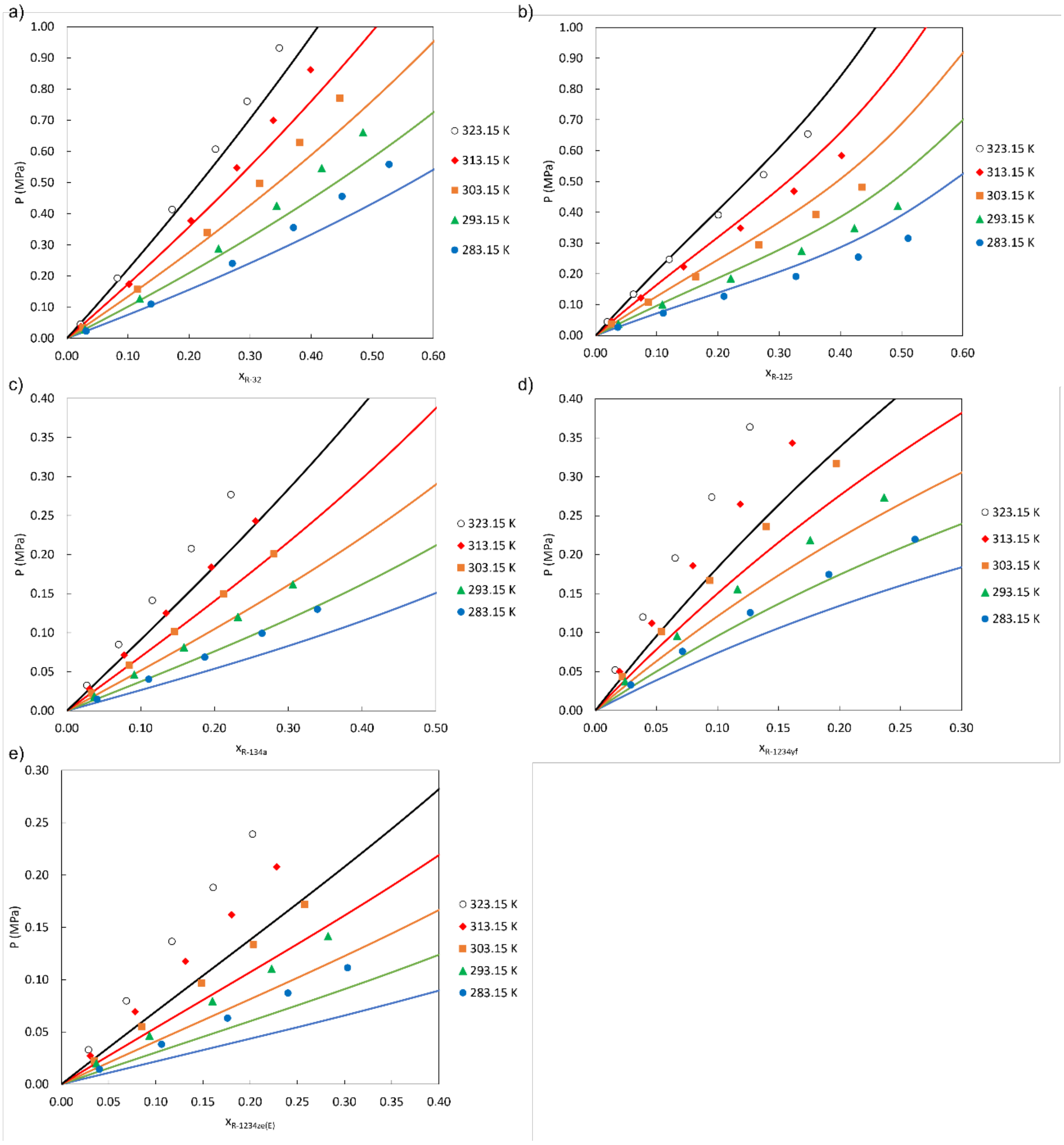

Figure S7. Solubility isotherms of: a) R-32, b) R-125, c) R-134a, d) R-1234yf, and e) R-1234ze(E), in RPC. The points are the experimental data, and lines represent the isotherms from COSMO-RS.

9. Henry law constants from experimental data and COSMO-RS

Table S18. Henry's law constants (MPa) of refrigerant gases in SKL.

Gas	Method	283.15 K	293.15 K	303.15 K	313.15 K	323.15 K
R-32	Experimental	1.999	2.685	3.461	4.580	5.443
	COSMO-RS	1.857	2.337	2.888	3.509	4.202
R-125	Experimental	2.144	3.035	4.074	5.422	7.394
	COSMO-RS	1.615	2.050	2.548	3.108	3.728
R-134a	Experimental	1.041	1.438	1.977	2.587	3.312
	COSMO-RS	0.614	0.815	1.056	1.340	1.668
R-1234yf	Experimental	2.208	3.127	3.982	5.170	6.527
	COSMO-RS	1.630	2.056	2.541	3.084	3.681
R-1234ze(E)	Experimental	0.855	1.253	1.603	2.165	2.946
	COSMO-RS	0.530	0.713	0.936	1.200	1.508

Table S19. Henry's law constants (MPa) of refrigerant gases in PC.

Gas	Method	283.15 K	293.15 K	303.15 K	313.15 K	323.15 K
R-32	Experimental	1.815	2.369	3.023	3.831	4.924
	COSMO-RS	1.841	2.316	2.862	3.482	4.174
R-125	Experimental	3.054	4.297	6.007	8.285	11.814
	COSMO-RS	3.319	4.043	4.843	5.715	6.654
R-134a	Experimental	1.190	1.639	2.201	2.892	4.014
	COSMO-RS	0.902	1.171	1.487	1.854	2.271
R-1234yf	Experimental	3.641	4.984	6.616	8.289	10.621
	COSMO-RS	3.489	4.229	5.040	5.918	6.857
R-1234ze(E)	Experimental	1.276	1.791	2.409	3.171	4.014
	COSMO-RS	1.021	1.327	1.687	2.103	2.575

Table S20. Henry's law constants (MPa) of refrigerant gases in TPN.

Gas	Method	283.15 K	293.15 K	303.15 K	313.15 K	323.15 K
R-32	Experimental	4.866	6.115	7.024	9.201	11.730
	COSMO-RS	3.937	4.749	5.647	6.630	7.695
R-125	Experimental	5.622	7.642	8.907	11.763	13.583
	COSMO-RS	2.065	2.541	3.075	3.666	4.314
R-134a	Experimental	2.759	3.616	4.389	5.681	7.054
	COSMO-RS	1.164	1.477	1.840	2.255	2.721
R-1234yf	Experimental	2.046	2.623	3.392	4.125	5.111
	COSMO-RS	1.049	1.325	1.642	2.002	2.404
R-1234ze(E)	Experimental	1.238	1.763	2.078	2.703	3.102
	COSMO-RS	0.560	0.736	0.947	1.195	1.482

Table S21. Henry's law constants (MPa) of refrigerant gases in GVL.

Gas	Method	283.15 K	293.15 K	303.15 K	313.15 K	323.15 K
R-32	Experimental	1.259	1.657	2.096	2.621	3.313
	COSMO-RS	1.152	1.482	1.871	2.320	2.833
R-125	Experimental	1.130	1.602	2.213	3.007	4.117
	COSMO-RS	1.138	1.465	1.846	2.283	2.775
R-134a	Experimental	0.634	0.898	1.115	1.484	1.942
	COSMO-RS	0.370	0.501	0.664	0.859	1.090
R-1234yf	Experimental	1.963	2.640	3.511	4.537	5.855
	COSMO-RS	1.422	1.805	2.245	2.743	3.298
R-1234ze(E)	Experimental	0.656	0.939	1.263	1.801	2.274
	COSMO-RS	0.370	0.506	0.674	0.877	1.118

Table S22. Henry's law constants (MPa) of refrigerant gases in RPC.

Gas	Method	283.15 K	293.15 K	303.15 K	313.15 K	323.15 K
R-32	Experimental	0.742	0.980	1.250	1.582	2.167
	COSMO-RS	0.979	1.259	1.587	1.966	2.398
R-125	Experimental	0.506	0.718	1.060	1.449	1.978
	COSMO-RS	0.660	0.862	1.101	1.380	1.698
R-134a	Experimental	0.346	0.491	0.670	0.903	1.186
	COSMO-RS	0.263	0.359	0.478	0.623	0.795
R-1234yf	Experimental	1.087	1.449	1.849	2.463	2.926
	COSMO-RS	0.815	1.049	1.322	1.635	1.988
R-1234ze(E)	Experimental	0.350	0.458	0.621	0.856	1.138
	COSMO-RS	0.222	0.308	0.416	0.547	0.705

10. Enthalpy and entropy of solvation

For the calculation of the enthalpy and entropy of solvation, the equations 34 and 35 were used:

$$\Delta H_{solv} = R \cdot \left(\frac{\partial \ln(k_H)}{\partial \left(\frac{1}{T}\right)} \right)_p \tag{S37}$$

$$\Delta S_{solv} = -R \cdot \left(\frac{\partial \ln(k_H)}{\partial \ln(T)} \right)_p \tag{S38}$$

Table S23. Enthalpy and entropy of solvation of each pair refrigerant gas/green solvent.

Gas	$Solvent$	$\Delta H_{solv}(kJ\ mol^{-1})$	$\Delta S_{solv}(J\ mol^{-1}K^{-1})$
R-32	SKL	-18.86	-62.48
	PC	-18.83	-62.33
	TPN	-16.44	-54.50
	GVL	-18.22	-60.29
	RPC	-19.93	-66.00
R-125	SKL	-23.25	-76.93
	PC	-18.83	-84.59
	TPN	-16.66	-55.06
	GVL	-24.46	-80.95
	RPC	-26.09	-86.30
R-134a	SKL	-22.12	-73.13
	PC	-21.75	-71.94
	TPN	-17.71	-58.62
	GVL	-20.86	-69.03
	RPC	-23.41	-77.42
R-1234yf	SKL	-20.35	-67.30
	PC	-20.19	-66.76
	TPN	-17.39	-57.50
	GVL	-20.75	-68.66
	RPC	-19.14	-63.28
R-1234ze(E)	SKL	-22.98	-76.04
	PC	-21.82	-72.13
	TPN	-17.29	-57.11
	GVL	-23.91	-79.07
	RPC	-22.67	-75.07

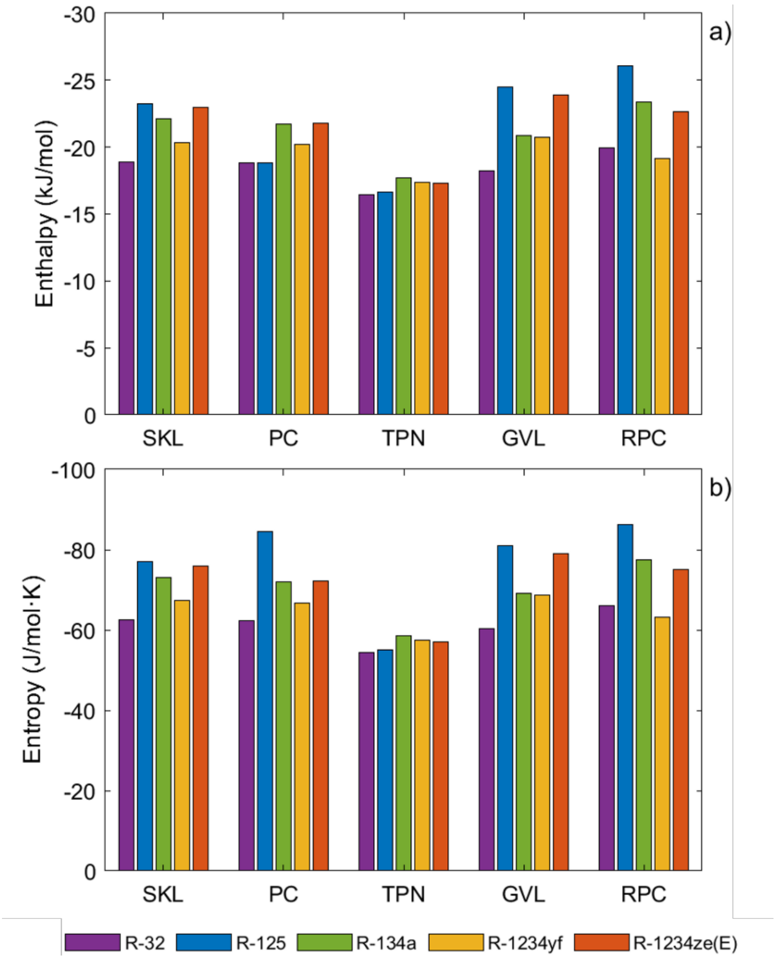

Figure S7. Enthalpy and entropy of solvation of F-gases in green solvents at 303.15 K.

11. Parametric analysis on the ARS performance

Additionally, a brief parametric assessment of the ARS performance was conducted to evaluate the influence of the temperature variables of the ARS, namely the generator, absorber, and evaporator. To this end, the low-GWP refrigerants HFC-32 and HFO-1234ze(E) were selected, in combination with the green solvent γ-valerolactone. The ARS configuration was based on the optimal scenario obtained in section 4.3 of the manuscript. Subsequently, Figure S8 illustrates the impact of each temperature on the COP values.

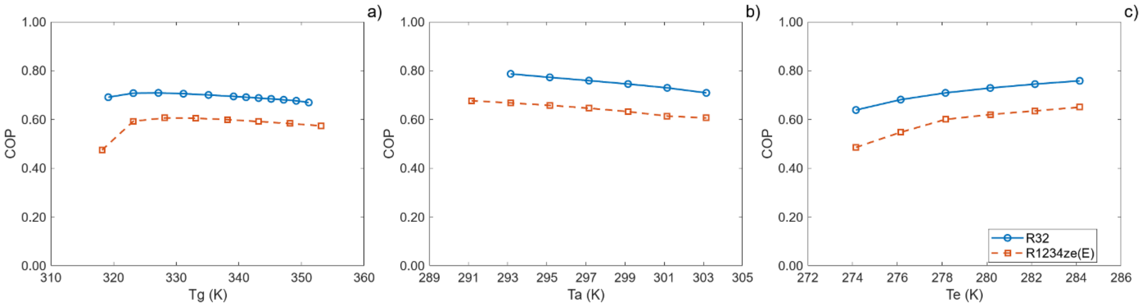

Figure S8. Influence of the temperature of a) the generator, b) the absorber, and c) the evaporator on the COP values for the best CA-ARS scenario (Table 4), obtained with pairs composed of R-32 or R-1234ze(E) as refrigerants, and γ-valerolactone as the solvent.

Regarding the generator temperature, its effect on the COP was negligible provided that a minimum temperature threshold was attained. This phenomenon was particularly evident in the case of R-1234ze(E), where a 20% decline in performance was observed at a temperature below 323.15 K. Conversely, above this temperature, further increases in temperature resulted in only a marginal reduction in the ARS performance. This outcome is advantageous, as acquiring waste heat to attain a temperature of 323.15 K is more feasible than reaching higher temperatures. The solubility of the refrigerant was found to be significantly influenced by the operating temperature of the absorber. As the operating temperature increases, the COP decreases because the difference in solubility between the solution leaving the absorber (stream 1) and that leaving the desorber (stream 4) is gradually smaller. However, this effect was mitigated by the practical constraints inherent to a typical refrigeration system. In relation to the evaporator, the primary objective is to achieve the desired cooling effect. A temperature range of 274.15 to 284.15 K was identified as hypothetical temperatures. It was observed that increasing the evaporator temperature led to an enhanced cycle performance, with the COP approaching 0.8 for R-32.

References

[1] E.D. Nikitin, A.P. Popov, N.S. Bogatishcheva, M.Z. Faizullin, Critical temperatures and pressures, heat capacities, and thermal diffusivities of γ-valerolactone and some alkyl pentanoates, J. Chem. Thermodyn. 149 (2020) 106162. https://doi.org/10.1016/j.jct.2020.106162.

[2] M. Shokouhi, H. Farahani, M. Hosseini-Jenab, Experimental solubility of hydrogen sulfide and carbon dioxide in dimethylformamide and dimethylsulfoxide, Fluid Phase Equilib. 367 (2014) 29–37. https://doi.org/10.1016/j.fluid.2014.01.020.

[3] D. Deng, G. Han, Y. Jiang, N. Ai, Solubilities of carbon dioxide in five biobased solvents, J. Chem. Eng. Data 60 (2015) 104–111. https://doi.org/10.1021/je500812s/.

[4] X. Liang, S. Ye, Q. Xie, M. Lu, F. Xia, Y. Nie, Z. Pan, J. Ji, Solubilities of sulfuryl fluoride in propylene carbonate, tributyl phosphate and N-methylpyrrolidone, J. Chem. Thermodyn. 125 (2018) 11–16. https://doi.org/10.1016/j.jct.2018.05.007.

[5] L.F. Lepre, D. Andre, S. Denis-Quanquin, A. Gautier, A.A.H. Pádua, M. Costa Gomes, Ionic Liquids Can Enable the Recycling of Fluorinated Greenhouse Gases, ACS Sustain. Chem. Eng. 7 (2019) 16900-16906. https://doi.org/10.1021/acssuschemeng.9b04214.

[6] J.E. Sosa, R.P.P.L. Ribeiro, P.J. Castro, J.P.B. Mota, J.M.M. Araújo, A.B. Pereiro, Absorption of Fluorinated Greenhouse Gases Using Fluorinated Ionic Liquids, Ind. Eng. Chem. Res. 58 (2019) 20769–20778. https://doi.org/10.1021/acs.iecr.9b04648.

[7] F. Murrieta-Guevara, A. Romero-Martinez, A. Trejo, Solubilities of carbon dioxide and hydrogen sulfide in propylene carbonate, N-methylpyrrolidone and sulfolane, Fluid Phase Equilib. 44 (1988) 105–115. https://doi.org/10.1016/0378-3812(88)80106-7.

[8] S. Asensio-Delgado, F. Pardo, G. Zarca, A. Urtiaga, Absorption separation of fluorinated refrigerant gases with ionic liquids: Equilibrium, mass transport, and process design, Sep Purif Technol 276 (2021) 119363. https://doi.org/10.1016/j.seppur.2021.119363.

[9] S. Asensio-Delgado, M. Viar, A.A.H. Pádua, G. Zarca, A. Urtiaga, Understanding the Molecular Features Controlling the Solubility Differences of R-134a, R-1234ze(E), and R-1234yf in 1-Alkyl-3-methylimidazolium Tricyanomethanide Ionic Liquids, ACS Sustain. Chem. Eng. 10 (2022) 15124–15134. https://doi.org/10.1021/acssuschemeng.2c04561.

[10] K.R. Baca, K. Al-Barghouti, N. Wang, M.G. Bennett, L. Matamoros Valenciano, T.L. May, I. V. Xu, M. Cordry, D.M. Haggard, A.G. Haas, A. Heimann, A.N. Harders, H.G. Uhl, D.T. Melfi,

A.D. Yancey, R. Kore, E.J. Maginn, A.M. Scurto, M.B. Shiflett, Ionic Liquids for the Separation of Fluorocarbon Refrigerant Mixtures, Chem. Rev. 124 (2024) 5167–5226. https://doi.org/10.1021/acs.chemrev.3c00276.

[11] I.H. Bell, J. Wronski, S. Quoilin, V. Lemort, Pure and Pseudo-pure Fluid Thermophysical Property Evaluation and the Open-Source Thermophysical Property Library CoolProp, Ind. Eng. Chem. Res. 53 (2014) 2498–2508. https://doi.org/10.1021/ie4033999.

[12] S. Asensio-Delgado, F. Pardo, G. Zarca, A. Urtiaga, Vapor−Liquid Equilibria and Diffusion Coefficients of Difluoromethane, 1,1,1,2-Tetrafluoroethane, and 2,3,3,3-Tetrafluoropropene in Low-Viscosity Ionic Liquids, J. Chem. Eng. Data 65 (2020) 4242–4251. https://doi.org/10.1021/acs.jced.0c00224.

[13] M.B. Shiflett, A. Yokozeki, Gaseous absorption of fluoromethane, fluoroethane, and 1,1,2,2-tetrafluoroethane in 1-butyl-3-methylimidazolium hexafluorophosphate, Ind. Eng. Chem. Res. 45 (2006) 6375–6382. https://doi.org/10.1021/ie060192.

[14] E.A. Finberg, T.L. May, M.B. Shiflett, Multicomponent Refrigerant Separation Using Extractive Distillation with Ionic Liquids, Ind. Eng. Chem. Res. 61 (2022) 9795–9812. https://doi.org/10.1021/acs.ierc.2c00937.

[15] K.S. Al-Barghouti, K.R. Baca, M.B. Shiflett, A.M. Scurto, Phase Equilibrium and Transport Properties of the Ionic Liquid 1-Ethyl-3-methylimidazolium Bis(trifluoromethylsulfonyl)amide and Compressed Difluoromethane and Pentafluoroethane, Ind. Eng. Chem. Res. 63 (2024) 1151–1169. https://doi.org/10.1021/acs.iecr.3c03491.

[16] A.M. Arishi, J.E. Espinoza Mejia, M.B. Shiflett, Separation of Azeotropic Refrigerant Mixtures: R-450A, R-456A, R-515B, and R-516A Using Phosphonium- and Imidazolium-Based Ionic Liquids, Ind. Eng. Chem. Res. 63 (2024) 6754–6765. https://doi.org/10.1021/acs.iecr.4c00531.

[17] W. Wu, H. Zhang, T. You, X. Li, Thermodynamic Investigation and Comparison of Absorption Cycles Using Hydrofluoroolefins and Ionic Liquid, Ind. Eng. Chem. Res. 56 (2017) 9906–9916. https://doi.org/10.1021/acs.iecr.7b02343.

[18] J.M. Asensio-Delgado, S. Asensio-Delgado, G. Zarca, A. Urtiaga, Analysis of hybrid compression absorption refrigeration using low-GWP HFC or HFO/ionic liquid working pairs, Int. J. Refrig. 134 (2022) 232–241. https://doi.org/10.1016/j.ijrefrig.2021.11.013.
